# Chemical, Physical, Microbial, and Sensory Properties of Innovative Sesame Milk Kefir, Focusing on the Ultrastructure of Kefir Grains

**DOI:** 10.1021/acsomega.4c08044

**Published:** 2025-02-18

**Authors:** Ibrahim A. A. Abou Ayana, Fatimah O. Al-Otibi, Mohamed R. Elgarhy, Mohamed M. Omar, Mohamed. Z. EL-Abbassy, Salah A. Khalifa, Yosra A. Helmy, WesamEldin I. A. Saber

**Affiliations:** † Dairy Research Department, Food Technology Research Institute (FTRI), Agricultural Research Center, Giza 12619, Egypt; ‡ Botany and Microbiology Department, Faculty of Science, King Saud University, Riyadh 11451, Saudi Arabia; § Food Science Department, Faculty of Agriculture, Zagazig University, Zagazig 44511, Egypt; ∥ Department of Veterinary Science, Martin-Gatton College of Agriculture, Food, and Environment, University of Kentucky, Lexington, Kentucky 40546, United States; ⊥ Microbial Activity Unit, Microbiology Department, Soils, Water and Environment Research Institute, Agricultural Research Center, Giza 12619, Egypt

## Abstract

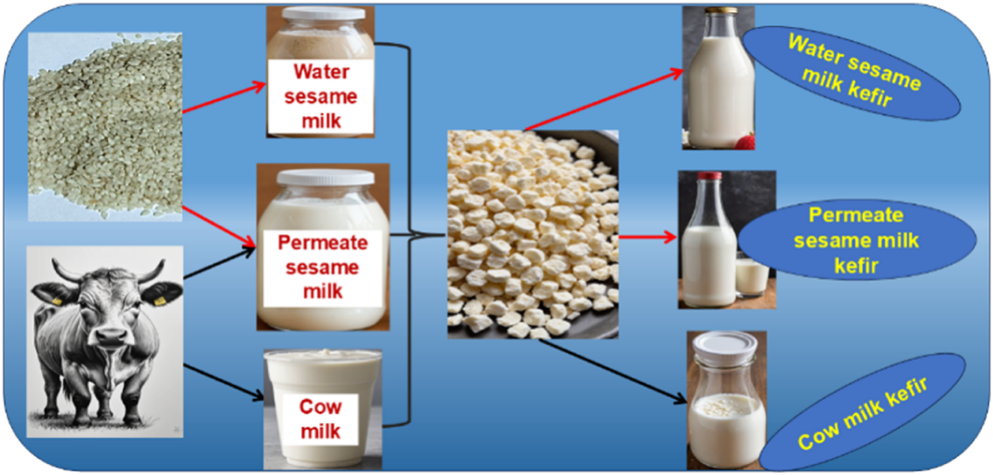

The demand for innovative plant-based probiotic beverages is growing rapidly. This study aimed to develop and evaluate a novel kefir beverage using two types of sesame milk: permeate-based sesame milk (PSM) and water-based sesame milk (WSM). Chemical, physical, microbial, and sensory properties of kefir were assessed. The total solids content (protein, fat, carbohydrates, and ash) in fresh kefir was 12.68, 13.31, and 16.38% for cow milk kefir (CMK), WSM kefir (WSMK), and PSM kefir (PSMK), respectively, and increased slightly after 14 days of storage, reaching 13.18, 13.53, and 16.56%. The fresh PSMK exhibited notable mineral content, containing (mg/100 g) 258.23 Ca, 137.14 P, 70.24 K, and smaller amounts of Na, Mg, Cu, Fe, Zn, and Mn, along with 5.18 μg/100 g of Se. In terms of volatile compounds, PSMK had the highest acetaldehyde concentration (7.48 mg/L), followed by CMK (4.91 mg/L) and WSMK (4.44 mg/L). Ethanol levels were the highest in fresh WSMK (0.129%). The viscosity and color attributes of PSMK were closely aligned with those of CMK, with the viscosity increasing over time to 1.53, 1.40, and 1.57 cP for PSMK, WSMK, and CMK, respectively. All kefir types supported viable probiotic populations, with PSMK demonstrating superior *Lactobacillus* and *Lactococcus* growth compared to WSMK. Sensory evaluations revealed high consumer acceptability for PSMK, comparable to CMK, with a purchase recommendation rate exceeding 76% for both PSMK and WSMK. Scanning electron microscopy revealed that the microstructure of PSMK grains was well-balanced and similar to that of CMK grains. This study highlights PSM as a promising dairy alternative for producing high-quality probiotic kefir, offering consumers an appealing, nutritious option within the growing plant-based beverage market.

## 1 Introduction

Plant-based milk alternatives have been consumed for centuries, providing valuable options for individuals with milk protein allergies, lactose intolerance, or those seeking dairy-free alternatives due to cholesterol-related health concerns, further providing a fermented product with high nutritional value to the vegetarianism group.^1^ These products aim to mimic animal milk in nutritional composition and functionality, providing proteins, amino acids, fats, fatty acids, vitamins, and minerals. While soy milk dominates the global plant-based milk market, there is growing interest in alternatives derived from various plants, including legumes, seeds, nuts, cereals, and pseudocereals.^2−4^

Sesame (*Sesamum indicum* L.) seeds, one of the oldest known oilseeds, have gained attention as potential sources of plant-based milk. Rich in oil (45.46–59.28%) and protein (21.43–25.77%), sesame seeds offer a promising nutritional profile for milk alternatives.^5,6^ The global production and harvested area of sesame have significantly increased over recent decades, reaching 7.4 million tons and 13.97 million hectares in 2022, respectively.^7^

The rising preference for plant-based milk alternatives is driven by various factors including health benefits, environmental concerns, dietary choices, and ethical considerations related to animal use. However, plant-based milk alternatives often fall short in nutritional value compared to animal milk and may suffer from limited shelf life, vegetal taste, and reduced consumer acceptability due to off-flavors.^2−4^

Fermentation has emerged as a promising approach to address these challenges in plant-based milk production. Kefir, one of the oldest fermented milk beverages, is produced by the synergistic action of a complex community of probiotic microorganisms, including various species of lactic acid bacteria (e.g., *Lactobacillus paracasei, Lactobacillus plantarum, Lactococcus lactis*), acetic acid bacteria (e.g., *Acetobacter* spp.), and yeasts (e.g., *Saccharomyces cerevisiae, Kluyveromyces marxianus*). These microorganisms are bound together within a polysaccharide matrix known as kefiran.^8,9^

Kefir offers numerous health benefits, including antiviral, antioxidative, anti-inflammatory, antimicrobial, anticancer, antidiabetic, and antiallergic properties.^10,11^ Regular consumption of kefir has been associated with improved digestive health, modulation of gut microbiota, increased lactose tolerance, and potential cardiovascular benefits.^11^

Kefir, a fermented dairy beverage renowned for its health benefits, is traditionally produced from cow or goat milk. As consumer preferences shift toward plant-based alternatives, there is a growing interest in developing kefir from dairy-free sources. Sesame milk, with its unique nutritional profile and rich flavor,^12^ presents a promising candidate for the creation of novel kefir formulations.

Sesame-based beverages are a valuable source of essential nutrients, such as vitamins, minerals, and dietary fiber. The lignans present in sesame seeds have been associated with various health benefits e.g., reduced inflammation, improved cardiovascular condition, and potential anticancer effects.^12^

Milk permeate, a byproduct of cheese production, offers an intriguing opportunity to enhance the nutritional composition of kefir. Rich in lactose and containing trace amounts of proteins, permeate is typically used for lactose production or as a spray-dried powder.^13,14^ Its composition includes 2–7% protein (mostly nonprotein nitrogen), 76–86% lactose, 0–1% fat, and 8–11% ash. However, a significant portion is often discarded or used in animal feed, leading to environmental and economic concerns. Valorizing permeate in kefir formulations can contribute to a more sustainable and resource-efficient food production system.^14−16^

Milk permeate has gained attention for its potential applications beyond traditional disposal methods in various nondairy food products, leveraging its nutritional profile.^17^ For instance, milk permeate has been utilized in the production of lactose-free, low-glucose, galactose-rich bioproducts through fermentation processes.^18^ Additionally, its incorporation into lipid-based nutrient supplements has shown promising results in improving the growth and body composition of undernourished children.^19^ These innovative applications highlight the value of milk permeate as a sustainable resource in the food industry.^17^

Despite the potential of sesame milk as a kefir base, limited research has focused on optimizing its formulation and fermentation process. Furthermore, the use of milk permeate in combination with sesame milk for kefir production remains an unexplored area with promising implications for both nutrition and sustainability. This study aims to investigate the feasibility of producing kefir from sesame milk and permeate, exploring the potential benefits in terms of nutritional value, sensory attributes, and microbial quality.

This study aims to develop a novel kefir beverage using sesame milk produced with permeate and evaluate its chemical, physical, microbial, and sensory properties in comparison to traditional cow milk kefir. Additionally, this research focuses on the ultrastructure of kefir grains grown in different milk types to better understand the impact of the fermentation medium on the microbial community development and kefir quality. By investigating this innovative approach to kefir production, we seek to address the growing demand for plant-based probiotic beverages while also exploring potential solutions for the utilization of dairy industry byproducts. This research contributes to the expanding field of functional, nondairy probiotic beverages and sustainable food production practices.

## 2 Material and Methods

### 2.1 Biotic Materials

Sweet white sesame (benniseed) seeds (*Sesamum indicum* L.), variety; Shandaweel 3, family; and Pedaliaceae, were thoughtfully provided from the Crops Research Institute, Agricultural Research Center, Giza, Egypt.

Standard cow milk (CM, 3% fat) was kindly obtained from the Dairy Department, Faculty of Agriculture, Mansoura University, Egypt.

Ultrafiltered milk permeate was gently provided by the Dairy Technology Unit, Dairy Department, Faculty of Agriculture, Mansoura University, Egypt. To protect permeate from microbial and chemical degradation, it was heat-treated at 80 °C for 10 min, then rapidly cooled to 4 °C, and stored frozen at −20 °C until required.

The spray-dried skimmed milk powder, low heat, of France origin was used during this work and was purchased from a private dairy plant in Zagazig, Egypt. Chemical characteristics butter fat; 0.5%, moisture; 3%, protein; 35.5%, lactose; 51.0%, minerals/ashes; 8.50%, titratable acidity; 0.15%, solubility index; 1.25 mL.

The culture of kefir grains (KG) was kindly provided by the Department of Food Engineering, Suleyman Demirel University, Isparta, Turkey, and maintained at 5 ± 2 °C until activated. By three consecutive successful fermentations, KG was activated using sterilized reconstituted milk (dry skim milk was dissolved in a specified amount of water to form liquid skim milk containing 11% total solids) at 25 °C/18 h, under normal atmospheric conditions, then strained to separate the KG to further studies.^20^

### 2.2 Sesame Milk Preparation

Two kinds of sesame milk were prepared using the previously proposed method;^12^ water-based sesame milk (WSM) and permeate-based sesame milk (PSM). Briefly, sesame seeds were washed and then soaked in water containing sodium bicarbonate (NaHCO_3_, 0.47%) for 11.24 h. Next, the sesame seeds were separated using cheesecloth. The two sesame milk types were prepared by blending water or milk permeate with the soaked sesame seeds at a ratio of 5 parts liquid to one part-soaked sesame seeds (5:1) at 25 °C, for 1.34 min before filtered using cheesecloth. Both sesame milk varieties were pasteurized (75 °C for 30 s) and refrigerated (4 ± 1 °C) until further investigation.

### 2.3 Preparation of Kefir Beverage

Various milk types were pasteurized at 90 °C for 15 min and cooled to 25 °C using water circulation by a heat exchanger. Cooled milks were inoculated with 5% (w/v) active KG and incubated (at 25 °C for 18 h). At the end of the incubation, KG were aseptically separated via a sterilized sieve and then the kefir beverage samples were taken into high-density polyethylene bottles.^21^ All kefir products were stored at 4 ± 2 °C for 14 days for further chemical and physical microbiological analysis as well as sensory evaluation.

### 2.4 Chemical Composition

The chemical composition of the biological materials was analyzed in three replicates. The contents of moisture, total solids, fat, total protein, ash, and titratable acidity were assayed as described by AOAC.^22^ Carbohydrates were estimated by subtracting fat, protein, and ash from the total solids. The pH value and acidity development of the various samples were measured at 25 °C using a digital pH meter (HANNA Instruments, 8417, Padua, Italy).

### 2.5 Minerals Contents

The content of iron, zinc, calcium, sodium, copper, selenium, phosphorus, and magnesium was detected by atomic absorption spectrophotometry (Varian Model Spectra AA 110 and 220), while potassium and manganese concentrations were detected using a Jenway PF7 Flame Photometer, Essex, UK.

### 2.6 Volatile Components of Kefir Beverage

#### 2.6.1 Acetaldehyde

Kefir beverage acetaldehyde content was determined based on the reaction of acetaldehyde with semicarbazide to form semicarbazone that has an absorption value at a wavelength of 224 nm, Results were expressed as milligram acetaldehyde per kg of kefir beverage.^23^

#### 2.6.2 Ethanol Content

Ethanol content in kefir beverages was determined using a modified dichromate method. Briefly, one mL of each kefir sample was placed in a 15 × 125 mm test tube and suspended in a 250 mL airtight flask containing 25 mL of potassium dichromate solution. Flasks were incubated at 25 °C for 48 h. Following incubation, 30 mL of distilled water was added to each flask, and the mixture was heated in a water bath at 62.5 °C for 20 min. After the mixture was cooled to room temperature, the absorbance was measured at 600 nm using a spectrophotometer. Ethanol concentrations were calculated based on a standard curve generated under the same conditions.^24^

### 2.7 Physical Measurements

#### 2.7.1 Viscosity

The milk viscosity was measured at 20 °C using a Brookfield digital viscometer (Model Number, Middleboro, MA 02346, U.S.A). Each sample was gently stirred five times in the clockwise direction with a plastic spoon to ensure homogeneity. Subsequently, the sample was placed in a small sample adapter, and a SC4–18 spindle was used. Viscosity measurements were recorded in centipoise (cP) at a rotational speed of 10 rpm for all samples.^25^

#### 2.7.2 Color

Color measurement was performed using a Hunter Lab Color Flex Model 45/0 instrument. Color data was recorded in CIE Lab color space, a standardized system where L* represents lightness (0–100), a* indicates the red-green axis (positive for red, negative for green), and b* represents the yellow-blue axis (positive for yellow, negative for blue). The instrument was calibrated using a white plate with reference values L* = 98.14, a* = −0.23, and b* = 1.89. The chroma value, a measure of color purity and intensity, was calculated using Eq (1)
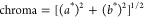
1To calculate the hue angle in degrees (*h*°) for different samples, the obtained values of the a* and b* were used as follows (eq 2). The negative *h*° value was summed with 180° to ensure the angle fell within the 0–360° range, allowing a precise comparison of the color.
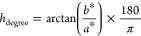
2

### 2.8 Microbiological Analysis

Experimental kefir beverage samples were subjected to microbiological analysis at fresh, 7-day, and 14-day intervals during cold storage. A variety of microbial groups (total viable bacteria, Lactobacilli, Lactococci, and yeast) were enumerated following these guidelines outlined in *Standard Methods for the Examination of Dairy Products*.^26^ All media used were supplied by Oxide Ltd., Basingstoke, UK. Tryptone water at 15 g/L was used to prepare the dilutions for the microbiological enumeration of tested microbial groups. Three replicates were used for each sample. The colony-forming units (cfu) were measured and expressed as logarithmic number (log) cfu g^–1^.

The total viable bacterial count was enumerated in one gram sample using the standard plate count technique after plating on nutrient agar medium, then incubated for 48 h at 30 °C.^27^

The Lactobacilli group was counted on MRS agar, a selective medium used for the cultivation of lactic acid bacteria (pH 6.2 ± 0.2). The incubation lasted for 3 days at 30 °C under anaerobic conditions using a gas pack (Oxide, UK) containing a mixture of 80% nitrogen, 10% carbon dioxide, and 10% hydrogen.^28^ Similarly, the lactococcal group was carried out on M17 agar, a selective medium designed for the enumeration of Lactococci (pH 6.9 ± 0.2). Plates were incubated for 2 days at 30 °C under anaerobic conditions using a gas pack (Oxide, UK).^29^ To inhibit yeast growth, 200 mg/L of cycloheximide was added to the two above-mentioned media.^30^

Yeast counts were enumerated^31^ in one gram of sample diluted in 9 mL of sterile solution of 2% (w/v) sodium citrate and homogenized in a Stomacher for 30 s to obtain 10-fold dilutions. Yeast was counted by surface plating on Yeast Potato Dextrose Agar with 0.01% chloramphenicol, after incubating at 25 °C for 3- 5 days.

### 2.9 Sensory Evaluation

The sensory evaluation of experimental kefir was carried out according to the methods of Lawless and Heymann^32^ and Abou Ayana and Saber^24^ with some modifications. A sensory panel of 20 dairy science specialists (8 females, 12 males; aged 25–54) was assembled to evaluate experimental kefir samples. Panelists were selected based on their experience in sensory evaluation of dairy products, particularly kefir and beverages. They were also required to have a keen sense of taste, smell, and appearance as well as a willingness to participate in sensory evaluation studies. Panelists were informed about the study’s objectives and provided written consent. Kefir samples (50 g) were presented in triplicate, coded with random numbers, and served chilled in transparent glass cups. A ranking scale (0–10 for appearance, color, smell, and taste; 0–5 for consistency, yeasty taste, refreshing taste, fermented taste, and purchase recommendation) was used to assess the sensory attributes. To ensure ethical rigor, the sensory evaluation adhered to the ethical guidelines of the Institute of Food Science and Technology.^33^

### 2.10 Studies on KG Biomass

#### 2.10.1 Weight of KG Biomass

Kefir beverages were prepared as mentioned earlier, and then, the growth of KG biomass (KGB) in various milk types was monitored in the fresh and during storage. The beverages, containing KGB, were stored without separating the KG. At specific time points (fresh and 7 and 14 days), KGB was extracted from each milk type, washed several times with sterile distilled water, and transferred to sterilized aluminum weighing dishes lined with sterilized paper towels to absorb excess moisture. The wet weight of the KGB was measured.^24^ The variation in KGB was calculated as follows (eq 3)
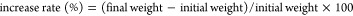
3

#### 2.10.2 Scanning Electron Microscopy (SEM)

KGB was fixed at 4 °C overnight in a buffered solution (1–2.5%) of glutaraldehyde + paraformaldehyde (2%) in sodium cacodylate buffer (0.1 M, pH 7.4). Following three 15 min rinses in cacodylate buffer (0.1 M) with sucrose (0.1 M), the KGB sample underwent postfixation in sodium phosphate buffered osmium tetroxide (2%, pH 7.4) for 90 min, with subsequent rinsing in 0.1 M cacodylate buffer. Dehydration was carried out using graded ethanol (50, 80, 90, and 96%) for 15 min each, followed by three 20 min immersions in 100% ethanol. The dehydrated sample was mounted on gold–palladium membranes, sputter-coated with a gold–palladium alloy, and examined using SEM (Jeol JSM-6510 L.V SEM) at 30 kV.

### 2.11 Statistical Analysis

All experiments were performed in triplicate (*n* = 3) at least, and data are presented as the mean ± standard deviation (SD). Statistical analyses were conducted using CoStat software (version 6.450, CoHort Software, Birmingham, UK). The treatments were arranged in a completely randomized design. One-way ANOVA was used to analyze differences among the various treatments. Following significant ANOVA results (α ≤ 0.05), Tukey’s Honestly Significant Difference (HSD) posthoc test was used for pairwise comparisons using minimum significant difference (MSD). Relationships between kefir beverages made from different milk types were assessed using Pearson’s correlation coefficient. All statistical tests were performed at a significance level of α ≤ 0.05.

## 3 Results and Discussion

This study investigates the nutritional properties, probiotic potential, consumer acceptance, and ultrastructural features of kefir produced from permeate-based sesame milk and water-based sesame milk, compared to traditional cow’s milk kefir. The study hypothesis was that there are no significant differences in the properties between the sesame milk kefirs and cow’s milk kefir. The alternative hypothesis states there are differences in at least one of these properties between the sesame milk kefirs and cow’s milk kefir.

### 3.1 Chemical Composition of Raw Materials

The final characteristics of a food product are largely determined by the chemical composition of the biomaterials used in its manufacture. Therefore, chemical analysis of the biomaterials used to produce kefir beverages is essential for this study. WSM, PSM, and CM in addition to the skim milk that was used to activate the KG were presented in Table 1. The standard cow’s milk (CM) with 3% fats, used in this study as a control, represents the typical milk used for kefir production.

**Table 1:** Compositional Characteristics of Water Sesame Milk (WSM), Permeate Sesame Milk (PSM), Cow Milk (CM), and Reconstituted Skim Milk^a^

					Tukey test	
criterion, %	cow milk	skim milk	WSM	PSM	*p*-value	MSD
total solids	11.32 ± 0.27	9.80 ± 0.15	12.87 ± 0.18	15.31 ± 0.73	0.000^b^	0.129
protein	3.39 ± 0.21	3.48 ± 0.13	2.85 ± 0.13	2.96 ± 0.12	0.000^b^	0.092
fat	3.02 ± 0.24	0.12 ± 0.07	5.34 ± 0.15	5.68 ± 0.20	0.000^b^	0.041
sugars	4.32 ± 0.20	4.67 ± 0.12	2.18 ± 0.08	5.62 ± 0.15	0.000^b^	0.047
fiber	0.00	0.00	0.72 ± 0.06	0.72 ± 0.05	0.000^b^	0.029
ash	0.63 ± 0.07	0.73 ± 0.01	0.48 ± 0.02	0.84 ± 0.01	0.000^b^	0.067
titratable acidity	0.16 ± 0.05	0.15 ± 0.05	0.14 ± 0.05	0.15 ± 0.06	0.389	ns
pH	6.64 ± 0.15	6.67 ± 0.23	6.75 ± 0.12	6.61 ± 0.15	0.000^b^	0.030

a Values represent mean ± SD (*n* = 3). One-way ANOVA was applied. Tukey’s HSD posthoc test was used for pairwise comparisons (α ≤ 0.05).

b Significant differences, ns; nonsignificant differences, MSD; minimum significant difference.

In general, PSM contained the highest percent of total solids (TS), fats, sugars, fibers, and ash compared to WSM and CM. The TS content in PSM compared to that in WSM, as observed in this study, is primarily attributed to the use of permeate in its production. Permeate, a byproduct of milk processing, is rich in TS, sugars, fibers, and ash, contributing to the increased solid content in PSM.

A potential factor contributing to an increase in ash content might be related to the moisture content. As water evaporates, the overall solid content, including ash, increases proportionally. Several studies have observed a slight rise in the ash content of kefir during storage.^34,35^ In our analysis, while significant differences in ash content were found among milk types, a minor increase in ash content over the storage period was not statistically significant (α ≤ 0.05) within each milk type.

Both WSM and PSM contained similar levels of fiber, at 0.72%. However, skim milk and CM had higher protein contents (3.48% and 3.39%, respectively) compared to PSM (2.96%) and WSM (2.85%). WSM exhibited lower fat content (5.34%) than previously reported values (7.09–8.02%).^36^

While the chemical composition of WSM can vary slightly, the TS content remains relatively consistent. This may be back to the roasting process that denatures some proteins, making them less soluble.^36^ Roasting softens cellulose, disrupting oil cells and promoting oil release during blending.^37^ Excessive cooking can also reduce milk fat content due to oil loss into the water.^38^

Comparing the two types of sesame milk, the ash content of PSM (0.84%) was higher than WSM (0.48%) and compared to previous reports.^36^ This difference between our results and the literature may be attributed to the blanching step, which can lead to mineral leaching into the blanching water.^39^

Beyond their nutritional value, kefir components significantly influence the final product’s properties, particularly its microbiota. As with any fermented food, the fermentation process is centered around microbes. The quality of the product is directly linked to the activity of these beneficial microbes, which, in turn, is influenced by the abundance of nutritional components in the fermentation medium.

TS plays a crucial role in microbial growth and product development. Higher TS levels, indicative of increased sugars, proteins, fats, minerals, and fibers, provide a more favorable environment for microbial proliferation. Sugars, in particular, are essential for the development of slight kefir acidity (pH and titratable acidity) during fermentation.^40^

Proteins contribute to the textural properties of kefir. The acid gel formed during milk fermentation by lactic acid bacteria creates a three-dimensional protein matrix that traps other milk substrates, including polysaccharides produced by the starter culture microflora. The nature and relative proportions of different proteins significantly influence the final product’s texture, as previously confirmed.^41^ Fats positively impact the sensory properties of kefir, contributing to its overall flavor and mouthfeel.^42^

Ash, which primarily consists of minerals, is essential for microbial metabolism and product quality. The presence of necessary minerals supports the growth and activity of beneficial microbes,^43^ ultimately influencing the overall characteristics of kefir.

### 3.2 Chemical Attributes of Kefir Beverage

Presently, there is a wide diversity of beverages from several plant sources.^44^ Fermentation is an ancient method for preserving, preparing, and improving the flavor and texture of foods and beverages depending on the enzymatic activity of the fermenting microbes. That produces proteins, amino acids, fatty acids, vitamins, organic acids, and volatile compounds, which gives the fermented products (such as yogurt, kefir, and cheese) distinctive tastes, flavors, and textures, as well as low cost.^45−47^ Fermentation of plant-based beverages differs depending on the composition of the certain plant matrix being fermented and the microorganisms used. Usually, lactic fermentation uses lactic acid bacteria such as *Lactobacillus* and *Streptococcus*, but *Saccharomyces cerevisiae* participates in alcohol fermentation.^46,48−51^

Fermentation is a promising method to enhance the flavor and taste of plant-based milk alternatives. This study conducted a comparative analysis of kefir beverages made from various milk types. The resulting kefir beverages were subjected to chemical, physical, microbiological, and sensory evaluations.

#### 3.2.1 Total Solids (TS)

Total solids content in kefir beverages varied based on the base milk used (Table 2). TS reflects the concentration of proteins, fats, carbohydrates, and minerals in kefir, influenced by the fermentation medium and its chemical composition. Fresh CMK had the lowest TS (12.68%) compared with WSMK (13.31%) and PSMK (16.38%). During cold storage, the TS values of all three types increased gradually to 13.18, 13.53, and 16.56%, respectively. The Food and Drug Administration requires fermented milk to contain at least 8.25% solids-not-fat, a standard commonly referenced in dairy regulations.^52^

**Table 2:** Chemical Composition Prepared by Different Milk Types during a Storage Period for 14 Days^a^

		component, %						
kind	time	TS	protein	fats	sugars	ash	TA	pH
CMK	fresh	12.68 ± 1.05	3.20 ± 0.20	3.10 ± 0.38	3.37 ± 0.35	0.66 ± 0.05	0.82 ± 0.02	4.62 ± 0.01
	7 day	12.98 ± 1.55	3.26 ± 0.25	3.11 ± 0.21	3.25 ± 0.16	0.69 ± 0.04	0.90 ± 0.02	4.52 ± 0.23
	14 day	13.18 ± 0.75	3.27 ± 0.11	3.10 ± 0.21	3.05 ± 0.18	0.71 ± 0.02	0.94 ± 0.07	4.45 ± 0.28
WSMK	fresh	13.31 ± 1.12	2.94 ± 0.15	5.24 ± 0.20	1.51 ± 0.25	0.76 ± 0.01	0.68 ± 0.05	4.75 ± 0.19
	7 day	13.42 ± 1.21	2.97 ± 0.25	5.27 ± 0.16	1.35 ± 0.25	0.78 ± 0.03	0.72 ± 0.02	4.71 ± 0.20
	14 day	13.53 ± 1.05	3.05 ± 0.28	5.26 ± 0.32	1.21 ± 0.18	0.82 ± 0.01	0.81 ± 0.07	4.63 ± 0.21
PSMK	fresh	16.38 ± 1.55	3.14 ± 0.15	5.89 ± 0.28	4.89 ± 0.92	0.84 ± 0.08	0.73 ± 0.03	4.70 ± 0.32
	7 day	16.42 ± 1.18	3.21 ± 0.12	5.91 ± 1.11	4.72 ± 0.55	0.91 ± 0.21	0.82 ± 0.11	4.63 ± 1.15
	14 day	16.56 ± 1.25	3.22 ± 0.15	5.90 ± 0.88	4.51 ± 0.65	0.94 ± 0.63	0.92 ± 0.49	4.46 ± 0.28
Tukey test	*p*-value	0.000^b^	0.009^b^	0.000^b^	0.000^b^	0.001^b^	0.000^b^	0.000^b^
	MSD	1.392	0.254	0.144	0.090	0.159	0.131	0.133

a TS; total solids, TA; titratable acidity. Values represent mean ± SD (*n* = 3). One-way ANOVA was applied. Tukey’s HSD posthoc test was used for pairwise comparisons (α ≤ 0.05).

b Significant differences, MSD; minimum significant difference.

The TS of seed sesame extract significantly influenced the TS of the experimental kefirs, with PSMK consistently having a higher TS than WSMK. A recent study demonstrated that the TS of probiotic products increases with higher proportions of plant-based milk.^53^ The increase in TS content observed in our stored sesame milk kefir aligns with previous findings that reported a significant increase in TS content in plant-based yogurts produced from Bambara nut, soybean, and *Moringa oleifera* seed milk types during storage.^54^

#### 3.2.2 Protein, Fats, and Ash

Kefir beverages made with CMK, PSMK, or WSMK exhibited varying levels of protein, fat, and ash (Table 2). CMK contained the highest protein content (3.2%), followed by PSMK (3.14%) and SMK (2.92%). All protein levels increased slightly during 14 days of storage. During fermentation, kefir grain microorganisms consumed sugars for energy, leading to a slight increase in protein content, possibly due to the production of microbial proteins by kefir grain yeasts. Fresh PSMK was rich in fats (5.89%), while WSMK was lower (5.24%) and CMK was the lowest (3.10%). Fat levels remained relatively stable throughout storage. PSMK also had the highest ash content, followed by WSMK and CMK. Due to moisture loss during storage, all kefir beverages recorded higher ash percentages after 14 days. These findings align with previous studies,^55^ but our results for PSMK are novel in this regard.

Fermenting plant-based drinks improves their nutritional value by breaking down complex molecules into simpler forms, making them easier to digest and absorb. This process enhances protein quality, increases the availability of nutrients, and may even produce additional vitamins.^56^

#### 3.2.3 Titratable Acidity and pH Values of Kefir Beverage

Despite cold storage, titratable acidity gradually increased in all of the kefir beverages (Table 2). This increase is primarily attributed to the metabolic activity of kefir grain microorganisms, which produce organic acids from lactose, or other sugars present in the kefir. The rate of acidity increases varied depending on the specific fermentable substrates available and the metabolic capacities of the diverse microbial communities within the KG.

CMK exhibited the most rapid acidity development, likely due to a combination of factors including a higher concentration of active microorganisms and a more favorable substrate composition. PSMK and WSMK had initially lower acidities, but their acidity levels increased significantly over the 14-day storage period.

The recorded acidity values, while relatively low, are consistent with the typical range for kefir products, especially considering the relatively short incubation and storage periods. The excess fat may hinder the development of acidity due to the accumulation of fat on the surface of the KG.^24,57^ This suggests that the acidity levels in our study are within the expected range for kefir-based products, and further highlights the importance of a diverse microbial community in kefir for optimal fermentation and acidity production.^8,9,12^

Regarding pH values, they decreased in the opposite direction of titratable acidity. This is a natural consequence of the increased concentration of organic acids. After 14 days of storage, the pH values of the control (CMK), WSMK, and PSMK dropped to 4.45, 4.63, and 4.46, respectively.

### 3.3 Mineral Content

Minerals are essential micronutrients that play crucial roles in human health. They are involved in various bodily functions, including maintaining blood pressure, immune function, bone health, and cell production. Macro-elements, required in amounts greater than 100 mg/day, and microelements, needed in smaller quantities, are both vital.^58,59^ However, tracking of the macro and microminerals of kefir after fermentation and during cold storage is an unprecedented study.

Regarding mineral analysis, a significant variation has appeared among analyzed items raw sesame seeds, unfermented WSM, PSM, and CM, and fresh or stored WSMK, PSMK, and CMK. The raw sesame seeds were macro- and microelements rich; this rich declined in both WSM and PSM (Table 3). However, the mineral content of kefir generally decreased slightly during fermentation. This reduction may be attributed to microbial activity during the kefir-making process.

**Table 3:** Macro-, and Microelemental Content in Various Kefir Beverages in Comparison to the Raw Biomaterials Used^a^

macro-element (mg/100 g)						
biomaterial		Ca	P	K	Na	Mg
sesame seeds		847 ± 5.12	6.21 ± 4.19	389.00 ± 5.10	2.37 ± 0.08	295 ± 5.06
milk	WSM	258.13 ± 1.53	172.54 ± 1.05	89.31 ± 0.92	65.78 ± 2.05	150.68 ± 2.25
	CM	121.13 ± 4.25	170.79 ± 4.17	111.21 ± 2.58	39.11 ± 0.23	13.92 ± 0.12
	PSM	290.45 ± 3.28	173.43 ± 2.18	102.51 ± 1.75	67.17 ± 1.37	159.51 ± 2.25
CMK	fresh	112.05 ± 1.17	160.24 ± 4.12	100.12 ± 1.98	37.37 ± 0.24	9.68 ± 0.05
	7 day	110.32 ± 3.18	151.08 ± 1.95	89.21 ± 0.86	37.12 ± 0.031	9.55 ± 0.02
	14 day	114.21 ± 3.11	121.62 ± 2.88	90.11 ± 0.83	35.12 ± 0.16	9.17 ± 0.21
WSMK	fresh	220.15 ± 1.58	121.05 ± 1.55	53.11 ± 1.07	40.27 ± 0.24	80.97 ± 0.48
	7 day	241.11 ± 2.17	112.31 ± 2.91	50.17 ± 1.54	38.18 ± 0.28	78.58 ± 0.38
	14 day	253.02 ± 1.55	132.70 ± 1.08	52.92 ± 2.01	44.19 ± 0.19	81.96 ± 0.29
PSMK	fresh	255.00 ± 0.14	133.21 ± 3.01	66.64 ± 0.93	44.18 ± 0.15	89.91 ± 1.12
	7 day	241.02 ± 0.15	125.11 ± 2.93	65.11 ± 0.35	44.01 ± 0.13	88.21 ± 1.34
	14 day	258.23 ± 0.08	137.14 ± 1.98	70.24 ± 1.22	45.00 ± 0.17	91.24 ± 0.28
Tukey test	*p*-value	0.000^b^	0.000^b^	0.000^b^	0.000^b^	0.000^b^
	MSD	6.05	4.26	3.10	4.37	3.71

microelement (mg/100 g)						
biomaterial		Cu	Fe	Zn	Mn	Se (μg/100 g)
sesame seeds		1.51 ± 0.040	13.9 ± 0.160	5.84 ± 0.25	1.250 ± 0.030	26.1 ± 0.37
milk	CM	0.02 ± 0.010	0.09 ± 0.020	0.37 ± 0.01	0.005 ± 0.001	1.02 ± 0.12
	WSM	0.77 ± 0.025	2.03 ± 0.120	3.51 ± 0.13	0.840 ± 0.120	3.21 ± 0.24
	PSM	4.77 ± 0.020	2.15 ± 0.030	3.55 ± 0.11	0.840 ± 0.020	6.77 ± 0.10
CMK	Fresh	0.01 ± 0.002	0.08 ± 0.001	0.33 ± 0.07	0.005 ± 0.001	1.01 ± 0.03
	7 day	0.01 ± 0.001	0.07 ± 0.004	0.31 ± 0.05	0.004 ± 0.002	1.00 ± 0.01
	14 day	0.01 ± 0.001	0.06 ± 0.003	0.30 ± 0.03	0.004 ± 0.001	1.02 ± 0.04
WSMK	Fresh	0.87 ± 0.010	1.50 ± 0.020	2.05 ± 0.05	0.410 ± 0.040	2.18 ± 0.18
	7 day	0.82 ± 0.030	1.21 ± 0.010	2.17 ± 0.18	0.430 ± 0.010	2.37 ± 0.13
	14 day	0.85 ± 0.040	1.28 ± 0.040	2.34 ± 0.13	0.540 ± 0.050	2.50 ± 0.08
PSMK	Fresh	3.51 ± 0.120	1.75 ± 0.010	2.34 ± 0.11	0.510 ± 0.020	5.12 ± 0.26
	7 day	3.24 ± 0.080	1.65 ± 0.030	2.22 ± 0.13	0.500 ± 0.040	5.00 ± 0.45
	14 day	3.47 ± 0.130	1.77 ± 0.050	2.45 ± 0.12	0.570 ± 0.020	5.18 ± 0.19
Tukey test	*p*-value	0.000^b^	0.000^b^	0.000^b^	0.000^b^	0.000^b^
	MSD	0.293	0.847	0.993	0.154	1.666

a Values represent mean ± SD (*n* = 3). One-way ANOVA was applied. Tukey’s HSD posthoc test was used for pairwise comparisons (α ≤ 0.05).

b Significant differences, MSD; minimum significant difference.

PSM was significantly richer in most minerals compared with WSM or CM, except for potassium. This enrichment is likely due to the high mineral content of sesame seeds, especially selenium, a vital nutrient.^60,61^ The permeate used in PSM production also contributes to its higher mineral content.

In our analysis, the kefir mineral content varied slightly during fermentation and storage. This variation may be due to the activity of kefir grain microorganisms. Additionally, the death and decomposition of microorganisms during fermentation may release certain elements into the kefir medium, contributing to the observed mineral changes. Fermentation enhances mineral bioavailability by degrading antinutritional factors such as saponins, tannins, phytic acid, and trypsin inhibitors that bind to minerals and hinder their absorption^62^

Ash (Table 2) is an indicator of mineral content, which increased during storage. However, specific mineral concentrations decreased, this discrepancy suggests additional, unidentified minerals and microbial activity influenced the final mineral composition.

### 3.4 Volatile Components

#### 3.4.1 Acetaldehyde

Acetaldehyde is a key compound contributing to the pungent and nutty aroma profile of fermented dairy products. Its formation, primarily catalyzed by the N-*streptococci* group, involves the conversion of lactose to glucose, which is subsequently metabolized via the Embden–Meyerhof–Parnas pathway into pyruvate. Pyruvate is then transformed into acetaldehyde through the action of α-carboxylase. Additionally, aldehyde dehydrogenase can convert acetyl-CoA (derived from pyruvate) into acetaldehyde.^63^

**Figure 1:**
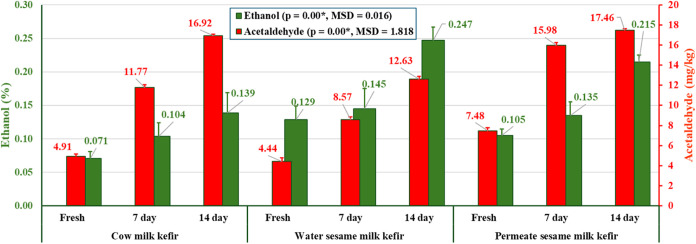
Volatile compounds in WSMK, PSMK, and CMK during the storage period. Values represent mean ± SD bare (*n* = 3). One-way ANOVA was applied. Tukey’s HSD posthoc test was used for pairwise comparisons (α ≤ 0.05). *Significant differences, MSD; minimum significant differences.

Figure 1 shows that the initial acetaldehyde levels were highest in PSMK (7.48 mg/L), followed by CMK (4.91 mg/L) and WSMK (4.44 mg/L). These concentrations significantly increased over 14 days, with PSMK consistently exhibiting the highest levels. A strong correlation between acetaldehyde production and lactose availability suggests that the higher lactose content in PSMK contributed to its elevated acetaldehyde formation. This finding aligns with the observation that PSMK outperformed CMK and WSMK in acetaldehyde synthesis. The acetaldehyde levels produced in our study were higher than those reported in kefir made with KG^64^ but lower than those typically found in other products,^65^ while the acetaldehyde concentrations align with those reported by Abou Ayana and Saber,^24^ This discrepancy is likely attributed to the metabolic action of alcohol dehydrogenase on alcohol molecules.^66^

#### 3.4.2 Ethanol

The production of volatile compounds in fermented dairy products is influenced by various factors, including microbial strain, incubation conditions, and milk composition. While ethanol levels are generally low in fermented milk, kefir stands out due to its unique microbial consortium, which includes yeasts. Consequently, kefir exhibits a significantly higher ethanol content compared to other fermented dairy products.

In this study, the type and composition of the fermentation medium emerged as the primary determinants of ethanol production (Figure 1). Ethanol concentrations in WSMK (0.129%) consistently exhibited the highest levels. The presence of readily fermentable sugars in sesame milk, such as fructose, glucose, raffinose, stachyose, and sucrose,^5,6^ likely contributed to the elevated ethanol production in WSMK.

Conversely, milk lactose, the primary sugar in CMK, is less readily fermented by yeast, the primary ethanol producer in kefir. The interplay between bacterial and yeast activity, influenced by the availability of sugars, resulted in moderate ethanol levels in PSMK.

Yeasts primarily colonize the surface of KG. The emulsified fat in cow milk kefir can hinder yeast activity by encapsulating KG and limiting nutrient availability.^8,9^ However, sesame milk’s fat composition appears to support higher yeast activity and ethanol production. While our findings show a higher ethanol content in WSMK compared to CMK, previous studies have reported varying ethanol levels in different kefir types.^24,67^ These inconsistencies may be due to chemical differences and microbial composition, processing conditions, and/or analytical methods.

### 3.5 Biophysical Features

#### 3.5.1 Viscosity

Viscosity expresses the thickness of a liquid; it affects the rheological and sensory properties of beverages. Thus, it has an important role in its acceptability by the consumer, as well as it is an important factor in determining the quality of fermented milk.

Table 4 presents the viscosity evolution of the studied kefir types over time. Fresh CMK, WSMK, and PSMK exhibited initial viscosities of 1.42, 1.33, and 1.45 cP, respectively. The inclusion of permeate in PSMK contributed to a viscosity profile similar to that of CMK. WSMK, characterized by a lower protein, sugar, fat, and ash content, displayed reduced initial viscosity. A slight moisture loss during storage led to a viscosity increase in all samples, with final values of 1.57, 1.40, and 1.53 cP for CMK, WSMK, and PSMK, respectively, after 14 days. This viscosity enhancement may positively impact the sensory perception of kefir, potentially resulting in a texture resembling that of CM-based kefir.

**Table 4:** Viscosity and Color Parameters of WSMK, PSMK, and CMK at Fresh and During Cold Storage^a^

			evaluation of kefir color				
sample	storage period	viscosity (cP)	L*	a*	b*	chroma	Hue angle (*h*°)
CMK	fresh	1.42 ± 0.17	82.11 ± 2.25	–4.101 ± 0.02	16.21 ± 0.55	16.72	104.16
	7 day	1.45 ± 0.14	81.18 ± 2.02	–4.621 ± 0.01	17.25 ± 0.35	17.86	104.96
	14 day	1.57 ± 0.15	81.15 ± 1.39	–4.688 ± 0.12	17.87 ± 0.48	18.47	104.66
WSMK	fresh	1.33 ± 0.17	79.97 ± 1.58	–4.89 ± 0.01	14.41 ± 0.99	15.22	108.71
	7 day	1.36 ± 0.12	77.11 ± 1.75	–6.293 ± 0.02	14.45 ± 0.87	15.76	113.50
	14 day	1.40 ± 0.13	76.05 ± 1.98	–6.381 ± 0.01	15.45 ± 0.56	16.72	112.41
PSMK	fresh	1.45 ± 0.11	81.06 ± 1.22	–4.091 ± 0.02	16.27 ± 1.11	16.78	104.08
	7 day	1.46 ± 0.13	80.21 ± 1.23	–3.168 ± 0.01	17.43 ± 1.15	17.72	100.26
	14 day	1.53 ± 0.09	81.18 ± 1.34	–3.171 ± 0.01	18.48 ± 1.18	18.75	99.70
Tukey test	*p*-value	0.037^b^	0.041^b^	0.000^b^	0.373		
	MSD	0.229	9.715	0.514	ns		

a Lightness (L*) ranged from 0 is black, and 100 is white. Red-green (a*) range from positive (red) and negative (green) values, 0 is neutral. Yellow-blue (b*), ranges from positive (yellow) and negative (blue) values, and 0 is neutral. Values represent mean ± SD (*n* = 3). One-way ANOVA was applied. Tukey’s HSD posthoc test was used for pairwise comparisons (α ≤ 0.05).

b Significant differences, ns; nonsignificant differences, MSD; minimum significant difference.

Viscosity is influenced mainly by the type and substrate of the fermentation. Acidification is the main factor of stable gel formation in milk, furthermore, milk composition, specifically protein and fat content contributes to higher viscosity.^68^ Microbial metabolites, particularly exopolysaccharides (EPS), influence the texture of fermented milk.^69^ Where, EPS can enhance the texture of fermented dairy products by binding water, interacting with proteins, and influencing viscosity and flow properties, with their production depending on the specific microorganisms and their growth conditions.^70,71^

#### 3.5.2 Kefir Color

The fresh innovative kefir made of PSMK showed a level of lightness (L*) 81.06 against 82.11, and 79.97 for CMK and WSMK. These mean that PSMK and WSMK head toward lightness. However, PSMK had L* greater than that of WSMK. CMK was more toward lightness 82.11. During cold storage, the lightness little decrease may be due to the concentration of total solids. L* reached 81.15, 81.18, and 76.05 for CMK, PSMK, and WSMK after 14 days (Table 4).

Color attributes a* (red-blue) of kefir samples were negative; all kefir showed yellowish (b*) color, and values of a* were negative, which indicates that it has taken toward the red color. PSMK was the highest in a*, which may be due to the reflected light on the sesame particles. WSMK was the lowest whether in fresh (−4.89) or after 14 days (−6.381). The color was poorly modified by fermentation, except for cow’s milk, for which fermentation increased to L* and b* values.^72^

Regarding b*, fresh WSMK was the lowest (14.41) and increased in PSMK (yellowish). This may be due to the presence of permeate in which more of riboflavin. CMK was moderated by a value of 16.21. b* took increasing direction with along storage (yellowish), the maximum value of b* was after 14 days for PSMK (18.48). The yellow color is attributed to riboflavin more than the fat effect.^73^

The lightness (L*) of fresh innovative kefir was 81.06 for PSMK, compared to 82.11 for CMK and 79.97 for WSMK, indicating that PSMK and WSMK were relatively lighter than CMK. However, PSMK exhibited a slightly higher lightness than WSMK. A slight decrease in L* was observed during cold storage for all samples, possibly due to increased total solids concentration. After 14 days, L* values were 81.15, 81.18, and 76.05 for CMK, PSMK, and WSMK, respectively.

All kefir samples displayed negative a* values, indicating a red-blue color shift toward red. PSMK had the highest a* value, potentially due to light reflection from sesame particles. WSMK exhibited the lowest a* values both initially (−4.89) and after storage (−6.381). Fermentation had a minimal impact on color, except for a slight increase in L* and b* for CMK.^72^

WSMK showed the lowest b* value (yellowness) initially (14.41), while PSMK had a higher b* due to increased riboflavin content in the permeate.^73^ CMK displayed a moderate b* value of 16.21. All samples experienced an increase in b* during storage, with PSMK reaching the highest value (18.48) after 14 days.

Table 4 presents the chroma and *h*° values of CMK, WSMK, and PSMK samples at the beginning of storage and after 7 and 14 days. Chroma, a measure of color saturation, showed an overall increasing trend for all samples during storage. An increase in chroma of fermented dairy products was reported during storage due to Maillard reactions and caramelization.^74^

The higher chroma in CMK could be due to the presence of fat-soluble pigments and other color-contributing compounds.^75^ The lower chroma in WSM might result from the absence of pigments, as sesame milk typically lacks natural colorants.^75^ The intermediate chroma in PSMK suggests that the permeate retains some color intensity, possibly due to the concentration of certain compounds during processing.^76^

Hue angle (*h*°), which indicates the color direction, exhibited variations among the samples. CMK maintained a relatively stable *h*° around 104°, suggesting a consistent color direction throughout storage. WSMK showed a slight increase in *h*° from 108.71 to 113.5° after 7 days, indicating a shift toward greener tones, which might be attributed to the presence of specific pigments or compounds in WSMK. PSMK displayed a more pronounced decrease in *h*° from 104.08 to 99.7° after 14 days, suggesting a shift toward redder tones. This trend of color formation could be a result of the combined action between sesame and permeate in PSMK.

A higher *h*° generally corresponds to lighter color perceptions. As *h*° increases, colors tend to shift toward yellow and green hues, often associated with a lighter appearance. In the context of kefir, elevated *h*° values suggest a movement toward a lighter tone (more yellowish). This color shift can be influenced by factors such as higher fat content and the presence of carotenoids, which are pigments that contribute to yellow coloration. Such visual attributes can enhance product appeal to consumers.^75^

It is important to note that the changes in chroma and *h*° were relatively small, indicating that the overall color of the kefir samples remained relatively stable during storage. However, further research is needed to investigate the specific factors influencing the color changes in these kefir types.

### 3.6 Microbial Pattern

Table 5 shows the behavioral pattern of microbial groups of kefir made from PSM or WSM compared to CM. As the total viable bacterial count expresses many bacteria capable of growing on standard, nonselective media, the total viable count was the highest. Furthermore, CMK contained the highest amount (8.53 log cfu g^–1^) followed by WSMK (7.42 log cfu g^–1^) and then PSMK (7.27 log cfu g^–1^) in fresh samples. After 7 days, these enumerations decreased to reach 7.66, 7.11, and 7.05 log cfu g^–1^, then slightly increased after 14 days, being 7.86, 7.31, and 7.18 log cfu g^–1^ for CMK, WAMK, and PSMK, respectively. The increment may be due to the adaptation of these bacteria in the different media. Furthermore, the change rate was the lowest in PSMK, and raised in WSMK then was higher in CMK.

**Table 5:** Total Viable, Lactobacilli, Lactococci, and Yeast Counts (log cfu g^–1^) in Fresh and during Cold Stored CMK, WSMK, and PSMK for 14 Days

		total bacteria		Lactobacilli		Lactococci		yeast	
type	time	count	change^a^	count	change^a^	count	change^a^	count	change^b^
CMK	fresh	8.53 ± 0.04		8.17 ± 0.58		8.26 ± 0.01		5.71 ± 0.08	
	7 day	7.66 ± 0.11	–0.87	7.65 ± 0.36	–0.52	7.46 ± 0.04	–0.8	5.77 ± 0.05	0.06
	14 day	7.86 ± 0.09	–0.67	7.19 ± 0.41	–0.98	6.86 ± 0.01	–1.4	5.81 ± 0.02	0.10
WSMK	fresh	7.42 ± 0.02		6.35 ± 0.02		6.69 ± 0.05		5.23 ± 0.01	
	7 day	7.11 ± 0.01	–0.31	5.77 ± 0.08	–0.58	5.88 ± 0.09	–0.81	5.41 ± 0.15	0.18
	14 day	7.31 ± 0.06	–0.11	5.51 ± 0.08	–0.84	5.64 ± 0.03	–1.05	5.46 ± 0.09	0.23
PSMK	fresh	7.27 ± 0.01		7.18 ± 0.05		7.22 ± 0.06		5.41 ± 0.01	
	7 day	7.05 ± 0.04	–0.22	6.75 ± 0.05	–0.43	6.63 ± 0.14	–0.59	5.49 ± 0.05	0.08
	14 day	7.18 ± 0.02	–0.09	6.41 ± 0.03	–0.77	6.21 ± 0.04	–1.01	5.57 ± 0.03	0.16
Tukey test	*p*-value	0.218		0.000^b^		0.000^b^		8.418	
	MSD	ns		0.323		0.829		ns	

a Change rate (%) relative to counts of the microbial group in the fresh sample. Values represent mean ± SD (*n* = 3). One-way ANOVA was applied. Tukey’s HSD posthoc test was used for pairwise comparisons (α ≤ 0.05).

b Significant differences, ns; nonsignificant differences, MSD; minimum significant difference.

Regarding Lactobacilli counts, CMK supported Lactobacilli growth whether fresh or stored samples, more than both types of sesame milk. However, PSMK promoted Lactobacilli growth more than WSMK. Lactobacilli counts decreased throughout the storage period, but the decline was higher in the first week than after that. Change rates in Lactobacilli were −0.98, −0.84, and −0.77 for CMK, WSMK, and PSMK, respectively; this may be due to sesame seed components that hinder the deterioration of Lactobacilli numbers, especially in the presence of permeate.

A similar pattern was reported during kefir storage, where until the second day of storage, the Lactobacilli count in the kefir was increased, then decreased until around the 14th day.^30^ Lactobacilli counts in kefir increased until the fourth day, which was then followed by a decline.^77^

CM promoted yeast growth more than either pattern of sesame milk, so fresh or stored CMK contained higher numbers against PSMK or WSMK. Unlike bacteria, whose numbers deteriorate during storage. The count of yeasts in all tested samples positively increased throughout the storage. In this connection, the counts of yeast are virtually constant up to 28 days of storage.^30^

WSMK achieved the highest rate of increase (0.18 and 0.23) against PSMK (0.08 and 0.16) and then CMK (0.06 and 0.10), after 7 and 14 days, respectively. Further, CMK contained higher yeasts count averse to WSMK or PSMK, the increase rate was higher in WSMK than PSMK. These results explain the raised concentration of ethanol in our WSMK or PSMK opposite to CMK. Sesame compounds may have activated the yeasts, despite their small number, and produced more ethanol than the effect of milk components. The yeasts count was different from the bacteria which decreased during refrigerator storage, but the yeasts tended to increase up to 14 days of storage in the following order: CMK > PSMK > WSMK.

### 3.7 Sensory Evaluation

**Figure 2:**
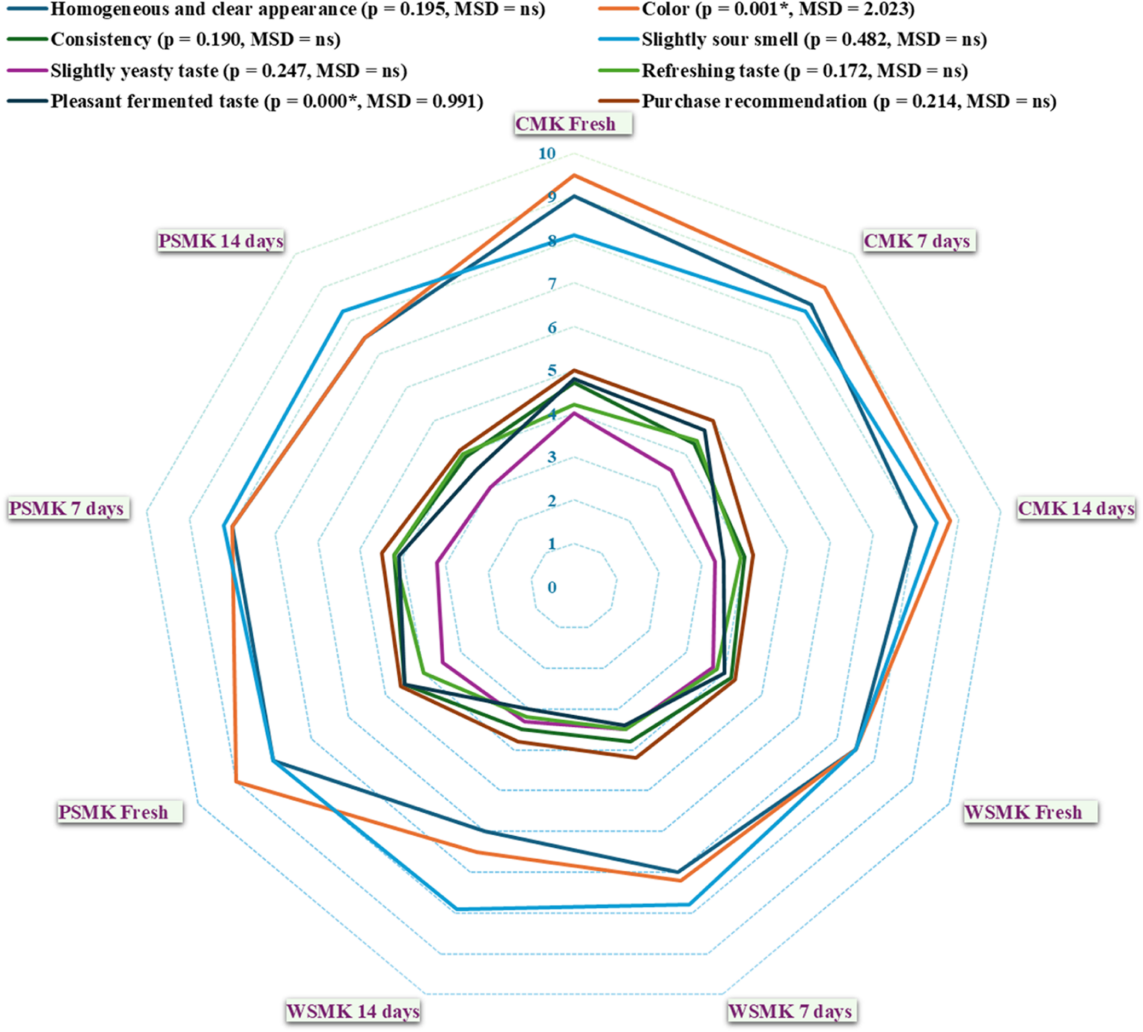
Sensory evaluation of WSMK, and PSMK compared to CMK during cold storage. Homogeneous and clear appearance, color, and slightly sour smell scored from 0 to 10, while consistency, slightly yeasty taste, refreshing taste, pleasant, fermented taste, and purchase recommendation scored from 0 to 5. Values are the mean of *n* = 20. One-way ANOVA was applied. Tukey’s HSD posthoc test was used for pairwise comparisons (α ≤ 0.05). *Significant differences, nanoseconds; nonsignificant differences, MSD; minimum significant difference.

Sensory evaluation is a crucial determinant of consumer acceptance of food products. Kefir’s sensory attributes reflect its chemical, physical, and microbial composition. In this study, CMK consistently received the highest level of consumer acceptance. PSMK exhibited comparable acceptance to CMK, while WSMK followed (Figure 2 and Table S1).

Most sensory evaluation determinants declined during cold storage, except for a slight increase in sour smell and refreshing taste after 7 days, followed by a minor decrease. Regarding Purchase recommendations, CMK, WSMK, and PSMK maintained high scores for 7 days but declined after 14 days, being 4.2/5, 3.8/5, and 4.1/5, respectively. Despite this, all sesame milk kefir types received recommendations from over 76% of panelists after 14 days, without significant differences among them, suggesting strong market potential.

This research marks the first evaluation of permeate-based sesame milk kefir. Unlike whey-based drinks, which often have a salty taste and cheesy odor,^78^ PSMK was highly acceptable. Previous studies observed surface mold formation after 14 days, leading to sensory evaluation termination.^30^ However, in our study, acceptance persisted for 14 days. While the highest sensory scores were awarded to CMK, the scores decreased as the plant-based component increased.^79,80^

To compare the sensory scores of the different kefir types more rigorously, the statistical analysis of Tukey’s HSD test was conducted. The results indicated nonsignificant differences (*p* < 0.05) among the three kefir types for several sensory attributes. However, CMK significantly outperformed WSMK and PSMK in terms of color, and pleasant, fermented. However, there were no significant differences between WSMK and PSMK for most sensory determinants.

The decline in sensory scores after 14 days of storage could be attributed to changes in the microbial composition, chemical reactions within the kefir matrix, and physical alterations such as whey separation or viscosity changes.^79,80^ These factors can collectively contribute to the evolution of flavor, aroma, texture, and overall sensory perception over time.

### 3.8 Correlation Study of Kefir Beverages

Table 6 presents the general correlation coefficients between kefir beverages made from different milk types. The analysis considered various tested factors during the investigated periods (Table S2). The results indicate a strong significant correlation between all three kefir beverages, with correlation coefficients ranging from 0.849 to 1.00 (*p* ≤ 0.05).

**Table 6:** General Correlation Coefficient of the Kefir Beverages Made from Different Milk Types^a^

Kefir beverage	CMK	WSMK	PSMK
CMK	1.000^b^		
WSMK	0.849^b^	1.000^b^	
PSMK	0.851^b^	0.996^b^	1.000^b^

a The analysis used all collected data along the various periods, including chemical composition, mineral content, volatile components, biophysical features, microbial pattern, and sensory evaluation.

b Significant Pearson’s correlation coefficient at α ≤ 0.05, cow milk kefir (CMK), water sesame milk kefir (WSMK), permeate sesame milk kefir (PSMK).

Furthermore, CMK shows a similarity to PSMK (0.851, *p* ≤ 0.05) than WSMK (0.849, *p* ≤ 0.05). This mathematical analysis confirms the similarity of our proposed kefir products to the natural CMK. However, both sesame kefir types showed a strong correlation with each other being 0.996, *p* ≤ 0.05, this further confirms the sesame seeds original of both WSMK and PSMK.

This suggests that the overall characteristics of these kefir beverages are highly similar, despite the differences in their milk base. This suggests that the fermentation process and microbial interactions play a more significant role in determining the final product’s properties than the specific type of milk used.

### 3.9 Kefir Grains Investigation

#### 3.9.1 Kefir Grains Biomass (KGB)

Key factors influencing kefir characteristics are the growth medium, kefir grain development, and fermentation conditions (temperature and duration). The chemical composition of the growth medium affects the microbial makeup of KG and kefiran, impacting kefir’s quality, biomass weight, and therapeutic properties.^24^ Therefore, measuring KGB was essential to assess the ability of KG to adapt and grow in different milk types, especially PSM. KGB was determined pre- and postincubation to evaluate the impact of various milk types on the growth rate of KG.

Table 7 shows the changes in KGB, pH values, and acidity of kefir in response to the milk type and storage period. Despite this, WSM supported the growth of KG but achieved the lowest increment of KGB (41.56%), against 49.60, and 55.51% for PSM and CM, respectively. The chemical composition of milk, e.g., carbohydrates, fats, and proteins (Table 1) greatly affected KGB. The high ratio of protein (3.42%) includes casein in addition to lactose in CM supplemented by the increase of KGB.

**Table 7:** Impact of Milk Type (CM, WSM, and PSM) on Wet Weight of Kefir Grain Biomass (KGB) Growth, as well as pH and Titratable Acidity Development^a^

		KGB			
Kefir type	time	weight (g)	pH	IR, %	titratable acidity (%)
CMK	before incubation	2.56 ± 0.14		6.61 ± 0.11	0.16 ± 0.18
	fresh	3.93 ± 0.23	55.5	4.23 ± 0.15	1.05 ± 0.19
	7 day	4.17 ± 0.16	62.9	4.12 ± 0.20	1.13 ± 0.07
	14 day	4.23 ± 0.25	65.2	4.03 ± 0.18	1.14 ± 0.13
WSMK	before incubation	2.55 ± 0.15		6.72 ± 0.18	0.14 ± 0.19
	fresh	3.61 ± 0.21	41.6	4.45 ± 0.11	0.92 ± 0.05
	7 day	4.03 ± 0.15	58	4.23 ± 0.31	1.04 ± 0.15
	14 day	4.11 ± 0.31	61.2	4.15 ± 0.21	1.11 ± 0.12
PSMK	before incubation	2.52 ± 0.15		6.64 ± 0.20	0.15 ± 0.16
	Fresh	3.77 ± 0.27	49.6	4.28 ± 0.08	0.99 ± 0.01
	7 day	4.05 ± 0.12	60.7	4.18 ± 0.14	1.05 ± 0.05
	14 day	4.16 ± 0.20	65.1	4.02 ± 0.11	1.15 ± 0.18
Tukey test	*p*-value	0.000^b^		0.000^b^	0.000^b^
	MSD	0.191		0.065	0.058

a Before incubation: measured directly after manufacturing, and before fermentation. Fresh: after manufacturing and fermentation for 18 h (mature product). KGB: wet weight of kefir grain biomass. IR%: increase rate. Values represent mean ± SD (*n* = 3). One-way ANOVA was applied. Tukey’s HSD posthoc test was used for pairwise comparisons (α ≤ 0.05).

b Significant differences, MSD; minimum significant difference.

Previous results supported this hypothesis that the increase in KGB is associated with the percentage of protein in milk.^24^ Milk with high protein content has led to the greatest increases in KGB. Additionally, the presence of casein has been shown to enhance both kefiran production and KGB growth.^81^

Furthermore, substrates own higher protein content produced higher KGB than those with low protein levels, the highest increase of KGB was found in KG grown in milk supplemented with whey protein.^82^ The KGB in cow and soy milk kefirs increased compared to their initial weight by 1.58- and 3.15-fold, respectively.^67^

The protein content may be related to the KGB increment. Furthermore, changes in the chemical composition of the fermentation medium, such as vitamins, minerals, and carbohydrates, may affect the KG metabolism.^83^ For instance, our data show that the increment of KGB was linked to the carbohydrate content of fermentation substrates, where CM and PSM contain higher levels of sugars (4.36 and 5.62%, respectively) than WSM (2.17%). Thus, the KGB of CMK and PSMK were more than the KGB of WSMK. Despite this association, it cannot be confirmed since soy milk containing (2.91% sugars) inoculated with KG, increased KGB by 30%.^24^ However, adding brown sugar to goat’s milk and cow’s milk reduced the KGB compared to the absence of brown sugar.^84^

In terms of acidity and pH, increased acidity and a decline of pH were associated with increased growth of KGB. CMK was richer in acidity by 1.05% versus 0.99% and 0.92% for PSMK and WSMK, respectively, in the fresh product. That is due to the availability of carbon sources such as lactose or sesame sugars. This association was not observed when comparing acidity and growth in soy and cow’s milk kefir production, cow milk kefir recorded higher acidity than soy milk kefir.^67^ However, with the progress of storage time, a slight reduction in pH and an increase in titratable acidity were observed, indicating the viability of KG during the storage period.

#### 3.9.2 Correlation Among KG Types

The general correlation coefficients between different kinds of KG were estimated (Table 8) to explore the degree of consanguinity. The analysis considered various factors during the investigation periods, including the weight of kefir grain biomass, pH, and titratable acidity (Table S3). The results indicate a strong correlation between all three types of KG, with significant correlation coefficients ranging from 0.997 to 1.00 (*p* ≤ 0.05), suggesting that the overall characteristics of the three KG are highly similar, despite the differences in their origin.

**Table 8:** General Correlation Coefficient of the Kefir Grains (KG) Made from Different Milk Types^a^

type	CM-KG	WSM-KG	PSM-KG
CM-KG	1.000^b^		
WSM-KG	0.997^b^	1.000^b^	
PSM-KG	0.999^b^	0.999^b^	1.000^b^

a The analysis used all previous data during the investigated period, including the weight of kefir grain biomass, pH, and titratable acidity

b Significant Pearson’s correlation coefficient at α ≤ 0.05, before incubation, cow milk kefir grains (CM-KG), water sesame milk kefir grains (WSM-KG), permeate sesame milk kefir grains (PSM-KG).

Furthermore, CM-KG shows a higher similarity to PSM-KG (0.999, *p* ≤ 0.05) than WSM-KG (0.997, *p* ≤ 0.05). Accordingly, the correlation coefficient confirms the high similarity between CM-KG from one side and the proposed two sesame KG types (WSM-KG, and PSM-KG). Another, both sesame kefir types showed a strong correlation with each other (0.999, *p* ≤ 0.05). These calculations suggest similar behavior of KG microbiota in the different milk types during the fermentation and thus supposed the suitability of the two sesame milk types as fermentation medium for kefir production, with no obvious differences with cow milk.

#### 3.9.3 Kefir Grain Morphology

Kefir grains, a symbiotic culture of microorganisms, exhibited distinct morphological characteristics when grown in different milk types (Figures S1–S4). The WSM-KG was observed to be larger and more irregular in shape compared to the PSM-KG and CM-KG, which appeared more compact and spherical. This morphological variation can be attributed to the unique nutrient composition and physicochemical properties of each milk type, influencing the growth and aggregation of KG.

The composition of KG also varied across the different milk types. Previous studies have shown that KG primarily consists of a protein matrix, which provides structural support and serves as a habitat for the microbial community. The type and concentration of proteins in the milk can influence the protein composition and structure of the resulting KG.^85,86^ In this study, WSM, which contains higher levels of plant-based proteins, may have contributed to the formation of a larger, more irregular KG.

Regarding the grain formation mechanism, *Kefiranofaciens, Lactobacillus*, and *Saccharomyces turicensis* initially undergo autoaggregation and coaggregation, forming small granules. This aggregation process is enhanced by a decrease in pH.^87^*Lactococci* contribute significantly to the formation of KG by producing exopolysaccharides that stabilize these granules and facilitate the development of a structured biofilm.^8^ Biofilm-forming organisms, such as *Lactobacillus kefiri, Pichia fermentans* HY3, and *Kluyveromyces marxianus* HY1 then adhere to these granules, developing thin biofilms.^9^ However, as kefir-associated yeasts, Lactococci, and Lactobacilli continue to coaggregate and integrate with these biofilm-covered granules, three-dimensional microcolonies are formed. The increasing cell density, along with the accumulation of milk components, further facilitates the formation of KG.^8,9^

The microbial composition of KG is a crucial factor in determining the functional properties and characteristics of the fermented beverages they produce. It is expected that the different milk types would have selective pressures on the microbial community within the KG. For example, the higher lactose content in cow milk may favor the growth of lactose-fermenting bacteria, while the unique fatty acid profile of sesame milk may influence the abundance of lipase-producing microorganisms. Furthermore, the presence of plant-based proteins in WSM and PSM could potentially support the growth of specific microbial groups that are adapted to utilize these substrates.

The study found that as casein levels increased, KG became more compact, and spherical, and produced less ethanol as in CM-KG, and PSM-KG. Conversely, WSM-KG was larger with relatively lower biomass, irregularly shaped, and produced more ethanol but less acetaldehyde. These findings suggest that sesame sugars primarily fuel ethanol-producing yeasts, while milk and whey proteins, particularly casein, influence the physical structure of KG.

#### 3.9.4 Ultrastructure of KG

The microstructure of KG grown in three milk kinds reflects the influence of milk type and its chemical composition on the microbial community and, consequently, the chemical composition of the fermented product.

##### 3.9.4.1. Kefir Grains of CM

**Figure 3:**
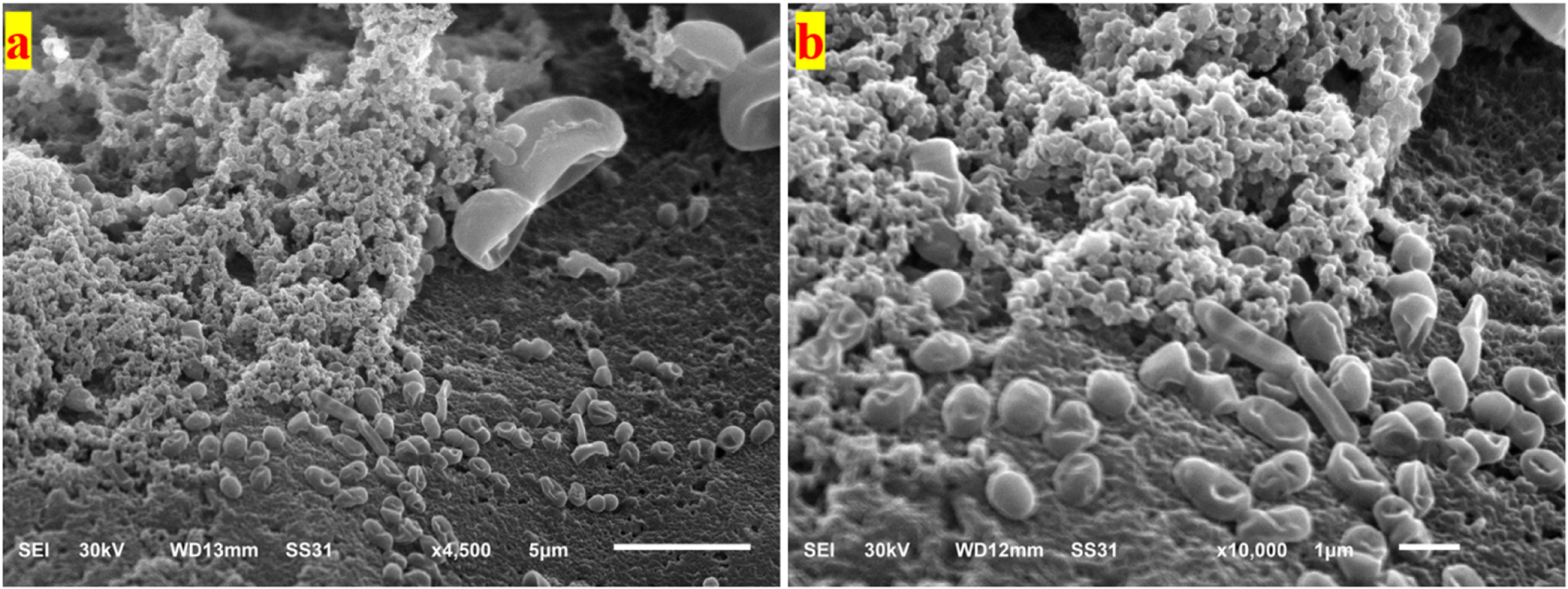
SEM image of kefir grain, developed in cow milk medium, showcasing the interconnected network of microbial cells within the kefiran matrix at a magnification of 4500× (a) and 10,000 (b).

The SEM images of CM-KG (Figures 3 and S5) revealed a diverse microbiota community, consisting of a variety of cell shapes, including cocci (spherical), bacilli (rod-shaped), and possibly other morphologies. The microbial cells were embedded within a matrix likely composed of kefiran, a polysaccharide produced by the symbiotic microorganisms within the KG.

The SEM image of CM-KG at a lower magnification (Figure 3a) showcased a dense, interconnected network of microbial cells, with a mix of spherical and rod-shaped cells evident. This arrangement suggests a healthy and well-established microbiota community, essential for the proper fermentation of milk and the production of kefir. This diversity likely contributes to the unique characteristics of kefir, including its probiotic properties and flavor profile. At higher magnification (Figure 3b), individual microbial cells were more discernible. While cocci and bacilli were visible, other cell shapes, such as curved bacteria, might also be present. The cells were tightly packed together, further emphasizing the dense nature of the microbial community. The matrix surrounding the cells appeared as a fine, filamentous network, providing a supportive structure for the diverse microbial population.

The fermentation medium’s chemistry impacted the KG structure, which then affected the final kefir. In our study of cow milk, bacteria (especially Lactobacilli and Lactococci) outgrew yeasts. This raised the acidity, lowered the pH, and increased the acetaldehyde. Sugar content also increased, possibly from kefiran-producing bacteria, boosting the viscosity. Alcohol decreased, likely because bacteria competed with yeasts, which struggle with lactose (cow milk’s only sugar). Based on these results, CMK, thus, received the best sensory scores.

##### 3.9.4.2. Kefir Grains of WSM

**Figure 4:**
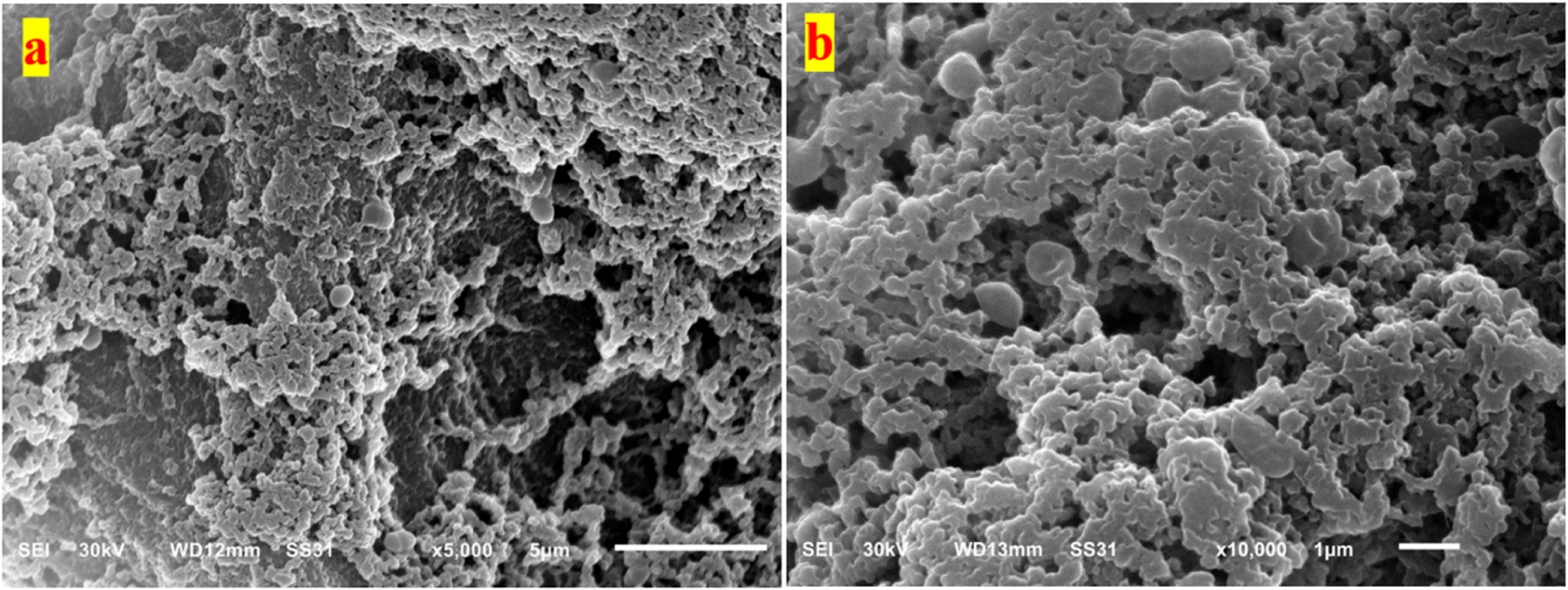
SEM image of kefir grain, developed in WSM medium, showcasing the interconnected network of microbial cells within the kefiran matrix at a magnification of 5000x (a) and 10000 (b).

The SEM images of WSM-KG (Figures 4 and S6) revealed a microbial structure like that observed in CM-KG. However, the microbial cells in WSM-KG appeared to be slightly less dense and less tightly packed (Figure 4a) than those in CM-KG. Additionally, the matrix surrounding the microbial cells seemed to be thinner and less prominent. Furthermore, the individual microbial cells in WSM-KG (Figure 4b) appeared to be less tightly packed together than in CM-KG, indicating a less supportive environment for the microbial community. The unique nutritional composition of WSM, containing only the sesame sugars (fructose, glucose, raffinose, stachyose, and sucrose), preferential supports the growth of yeasts over bacteria.^88^ This imbalance in microbial populations could influence the growth and metabolic activities of the KG microbiota. So, SMK had lower acidity, acetaldehyde, and viscosity but higher alcohol. This likely happened because SMK’s sugars better support yeast growth, which yeasts can easily use. These factors led to SMK getting the lowest sensory scores.

##### 3.9.4.1. Kefir Grains of PSM

**Figure 5:**
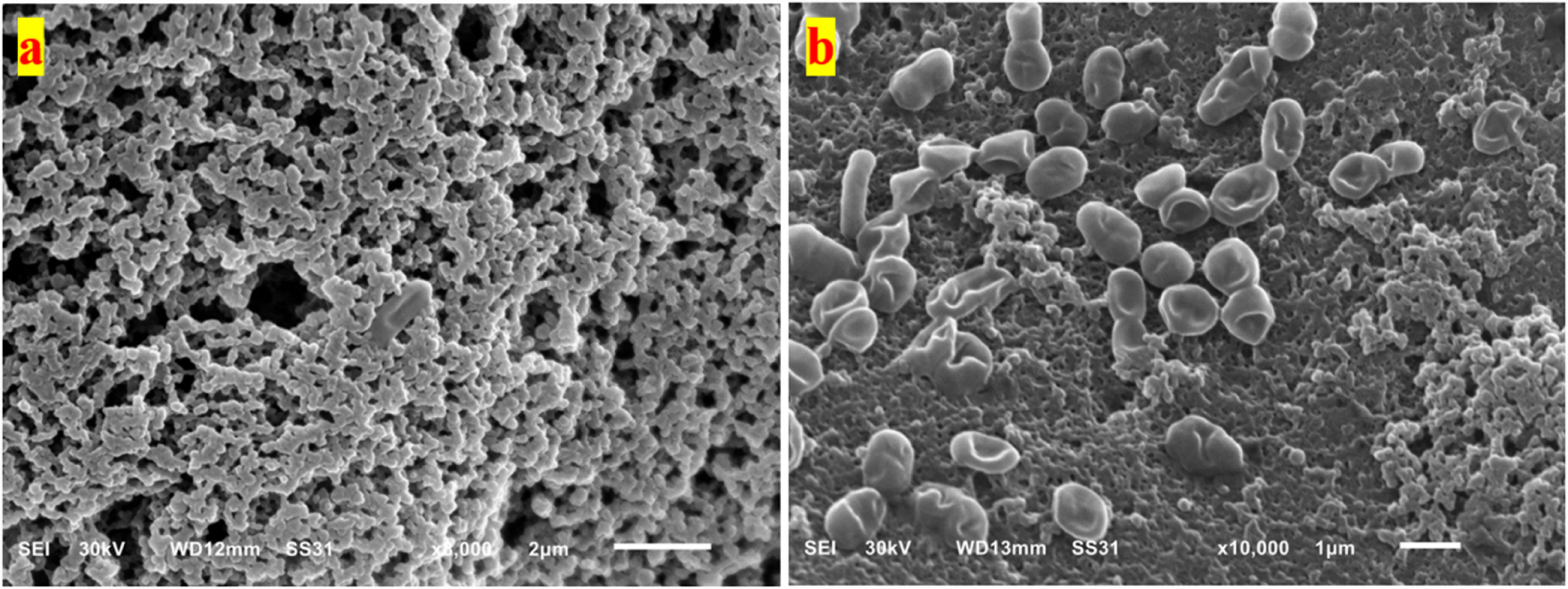
SEM image of kefir grain, developed in PSM medium, showcasing the interconnected network of microbial cells within the kefiran matrix at a magnification of 8000× (a), and 10,000 (b).

The SEM images of PSM-KG (Figures 5 and S7) revealed a microbial structure like that observed in CM-KG and WSM-KG. However, there were some notable differences. Figure 5a reveals a microbial structure that is intermediate between CM-KG and WSM-KG. The cells appear to be slightly less dense than in CM-KG but more densely packed than in WSM-KG. The matrix surrounding the cells is thicker than in WSM-KG but thinner than that in CM-KG. At a higher magnification (Figure 5b), individual microbial cells in PSM-KG can be observed. The cells are moderately packed together, and the matrix surrounding them is relatively thin. The presence of both lactose (from permeate) and sesame sugars suggests a more balanced environment for microbial growth compared to WSM-KG. This balanced nutrient composition may promote the growth of both bacteria and yeasts, as well as the production of kefiran.

The permeate in PSM promoted kefir grain microbiota growth, similar to that in cow milk. Consequently, PSMK’s acidity, pH, and protein content resembled CMK’s. However, PSMK contained more total solids, sugars, and fats due to PSM’s higher initial levels. This stable combination of sesame and permeate resulted in PSMK having the highest acetaldehyde concentration, moderate alcohol and viscosity, and sensory scores comparable to those of CMK.

In general, the KG developed in all three milk types exhibited similar textural and microbial structures with a diverse community of bacteria and yeast embedded within a matrix. However, notable differences were observed in the density, arrangement, and appearance of the microbial cells and surrounding matrix. These differences, likely influenced by the milk type, may impact the fermentation process and the final properties of the kefir beverage.^85,86^

Despite the observed spatial distribution of microorganisms within KG, with Lactococci and yeasts concentrated in the outer layers and Lactobacilli and additional yeasts in the inner layers, the symbiotic relationship among these KG microbiota remains crucial for their survival and the production of bioproducts, which drive lactic and alcoholic fermentation.^89,90^ This microbial community plays a crucial role in the production and characteristics of kefiran, a polysaccharide produced by symbiotic microorganisms within the grains. A more diverse microbial community may lead to higher kefiran production, while specific microbial species can influence the structural characteristics of kefiran. Additionally, interactions between different microorganisms within the KG community could affect the physical, chemical, and sensory properties of kefiran.^85,86,91^

To gain a deeper understanding of the microbial role in kefiran development by PSM, further studies could employ metagenomic analysis to identify microbial species involved in kefiran production, kefiran extraction, and characterization to analyze its properties, and microbial culture experiments to assess individual species’ kefiran-producing capabilities.

Despite the apparent similarities observed in the microbial structures of KG developed in different milk types, PSM appears to be a more suitable substrate for kefir production compared to WSM. The structure of PSM-KG is more similar to the standard CM-KG, suggesting that the presence of milk permeate in PSM may provide a more favorable environment for the growth and development of the kefir grain microbiota.^85,86^ This similarity in structure may lead to a kefir beverage with properties closer to those of traditional cow milk kefir.

Overall, the SEM images reveal distinct microstructures that correlate with microbial communities. CM-KG exhibits a dense interconnected network, suggesting a well-established microbial community. This dense structure corresponds to the highest initial microbial counts observed in CM-KG, indicating that this matrix provides a favorable environment for initial growth. In contrast, WSM-KG displays more loosely packed cells and a thinner matrix, correlating with lower microbial counts compared to those of CM-KG. This suggests a less supportive microbial environment. The matrix itself, likely composed of kefiran, influences moisture and nutrient retention, which directly impacts microbial activity and viability. Over the 14-day storage period, microbial counts decreased in all samples but CMK consistently maintained higher counts than both WSMK and PSMK, demonstrating better long-term viability in the denser CM-KG structure. However, the accurate microbiological constitution of KG is still controversial. More than 50 various bacterial and yeast species have been found in KG, the geographical origin of the KG and the cultivation conditions may largely influence the dynamics, and microbial composition.^21,92−94^

Finally, although PSMK kefirs and CMK are comparable, the study accepts the alternative hypothesis, suggesting some differences in several properties, such as nutritional and microbial features and ultrastructure, between the sesame milk kefirs and cow’s milk kefir.

### 3.10 Potential Limitations of the Study

This study, while offering valuable insight into sesame milk kefir, has some limitations. The small sample size and region-specific consumer evaluation limit the generalizability of the findings. Future research should include larger, more diverse consumer panels to better understand the broader acceptance.

Storage conditions and their impact on microorganism viability are key limitations. The 14-day refrigerated storage period does not reflect real-world storage scenarios or predict long-term stability. Fluctuations in temperature during storage, even within refrigeration, can significantly affect microbial survival and activity. For example, even brief temperature increases can accelerate metabolic processes, leading to a faster depletion of nutrients and a decline in viable cell counts. Conversely, excessively low temperatures (without proper cryoprotection) can cause cold shock and cell damage. Future studies should explore extended storage periods and implement more rigorous temperature control, including monitoring temperature fluctuations within the refrigerator. This would provide a more accurate assessment of the product quality and microbial viability over time. Investigating the long-term survival of probiotic microorganisms is crucial, as their metabolic activity and viability are essential for the claimed health benefits of kefir. Reduced activity or cell death during extended storage could diminish the product’s probiotic efficacy. Studies should employ methods such as flow cytometry or plate counting at various time points during storage to track viable cell counts and assess the impact of different storage conditions on specific probiotic strains.

Variability in raw materials (sesame and cow milk) due to the source, processing, and season could influence the results. Standardizing these materials in future research will improve consistency. Despite using the standard kefir inoculum, the study also focused on specific probiotic strains, not the full microbial diversity. Further investigation of microbial interactions is needed.

Finally, while nutritional components were assessed, bioavailability and health benefits in humans were not evaluated. Future research should consider clinical trials. Economic factors, such as production costs compared to dairy kefir and the potential for sesame allergies, also warrant further consideration.

## 4 Conclusions

This study successfully developed a novel kefir beverage using PSM, demonstrating its potential as a sustainable and nutritious alternative to traditional dairy kefir. PSMK exhibited comparable chemical, physical, and microbial properties to those of CMK, surpassing those of WSMK in several key areas. The addition of milk permeates to sesame milk created a more favorable environment for kefir grain growth, resulting in improved microbial proliferation and product quality. One of the most significant findings of this research was the similarity between PSMK and CMK in terms of viscosity, color, and sensory attributes. This suggests that PSMK can effectively replicate the texture, taste, and overall experience of traditional dairy kefir, making it a suitable option for consumers seeking plant-based alternatives. Furthermore, PSMK demonstrated levels of essential minerals and bioactive compounds comparable to those of CMK, highlighting its nutritional value. The presence of viable probiotic populations in PSM kefir further supports its potential health benefits. Scanning electron microscopy revealed that the microstructure of PSMK grains was well-balanced and, like that of CMK grains, characterized by a dense, interconnected network of cells and extracellular matrix. This similarity in microstructure suggests that PSMK may possess functional properties similar to those of CMK, such as texture and stability. While this study provides valuable information about the development and characterization of PSMK, future research is needed to explore its long-term stability, specific health benefits, and the dynamics of the microbial community within the kefir grains. Investigating the potential interactions between the unique components of PSM and the kefir microbiota may offer further insights into the mechanisms underlying the observed improvements in product quality. Additionally, conducting clinical trials to assess the health benefits of PSMK consumption would be essential to solidifying its position as a viable and beneficial plant-based probiotic beverage.

## Supporting Information Available

The supplementary data to this article contains Tables S1–S3 and Figures S1–S6. Table S1. Sensory evaluation of water sesame milk kefir (WSMK) and permeate sesame milk kefir (PSMK) compared to cow milk kefir (CMK) during cold storage. Table S2. The general arrangement of collected data obtained during kefir beverage preparation used for the estimation of the correlation coefficient between the three kefir beverages irrespective of the storage period. Table S3. The general arrangement of collected data of kefir grains used for estimation of the correlation coefficient between the three kefir grains irrespective of the storage period. Figure S1. Zoom-in view of kefir grain morphology developed in cow milk (a, and b), water sesame milk (c, and d), and permeate sesame milk (e, and f). These photos were taken by one of the authors during the manufacturing in the laboratory. Figure S2. Zoom-in view of kefir grain morphology developed in cow milk. These photos were taken by one of the authors during the manufacturing in the laboratory. Figure S3. Zoom-in view of kefir grain morphology developed in water sesame milk. These photos were taken by one of the authors during the manufacturing in the laboratory. Figure S4. Zoom-in view of kefir grain morphology developed permeate sesame milk. These photos were taken by one of the authors during the manufacturing in the laboratory. Figure S5. SEM image of kefir grain, developed in cow milk medium, showcasing the interconnected network of microbial cells within the kefiran matrix at magnification of 4500× (a), and 10,000 (b). Figure S6. SEM image of kefir grain, developed in WSM medium, showcasing the interconnected network of microbial cells within the kefiran matrix at magnifications of 5000× (a) and 10,000 (b). Figure S7. SEM image of kefir grain, developed in PSM medium, showcasing the interconnected network of microbial cells within the kefiran matrix at a magnification of 8000× (a), and 10,000 (b) (PDF)
