# Supplementary material for: Chemical, Physical, Microbial, and Sensory Properties of Innovative Sesame Milk Kefir, Focusing on the Ultrastructure of Kefir Grains
Source: ACS Omega. 2025 Feb 18;10(8):7752–69. doi: 10.1021/acsomega.4c08044 (PMC11886653; doi:10.1021/acsomega.4c08044)
Supplement: Supplementary file 1 — ao4c08044_si_001.pdf [file ao4c08044_si_001.pdf]

Supporting Information

Chemical, Physical, Microbial, and Sensory Properties of Innovative Sesame Milk Kefir, Focusing on the Ultrastructure of Kefir Grains

Ibrahim A.A. Abou Ayana [a,*], Fatimah O. Al-Otibi [b], Mohamed R. Elgarhy [a], Mohamed M. Omar [c], Mohamed. Z. EL-Abbassy [c], Salah A. Khalifa [c], Yosra A. Helmy [d], and WesamEldin I. A. Saber [e,*]

[a] Dairy Research Department, Food Technology Research Institute (FTRI), Agricultural Research Center, Giza 12619, Egypt; abouayana@gmail.com (IAA).

[b] Botany and Microbiology Department, Faculty of Science, King Saud University, Riyadh 11451, Saudi Arabia

[c] Food Science Department, Faculty of Agriculture, Zagazig University, 44511, Egypt

[d] Department of Veterinary Science, Martin-Gatton College of Agriculture, Food, and Environment, University of Kentucky, Lexington, KY 40546, USA

[e] Microbial Activity Unit, Microbiology Department, Soils, Water and Environment Research Institute, Agricultural Research Center, Giza 12619, Egypt; wesameldin.saber@arc.sci.eg

*Corresponding Authors: abouayana@gmail.com (IAA), wesameldin.saber@arc.sci.eg (WIAS)

Table S1. Sensory evaluation of water sesame milk kefir (WSMK) and permeate sesame milk kefir (PSMK) compared to cow milk kefir (CMK) during cold storage.

Type	Storage	Sensory evaluation determinant								Total score/50	Percentage
		Homogeneous and clear appearance (0-10)	Color 0-10	Consistency (0–5)	Slightly sour smell (0–10)	Slightly yeasty taste (0–5)	Refreshing taste (0–5)	Pleasant, fermented taste (0–5)	Purchase recommendation (0–5)		
CMK	Fresh	9.0±0.35	9.5±0.25	4.7±0.20	8.1±0.51	4.0±0.12	4.2±0.10	4.8±0.15	5.0±0.10	49.3	98.6
	7 day	8.5±0.31	9.0±0.22	4.3±0.15	8.3±0.25	3.5±0.08	4.4±0.15	4.7±0.08	5.0±0.12	47.7	95.4
	14 day	8.0±0.41	8.8±0.26	4.0±0.22	8.5±0.15	3.3±0.15	3.9±0.08	3.5±0.05	4.2±0.15	44.2	88.4
WSMK	Fresh	7.5±0.15	7.5±0.35	4.2±0.26	7.5±0.11	3.7±0.04	3.8±0.13	4.0±0.10	4.3±0.14	42.5	85.0
	7 day	7.0±0.27	7.2±0.18	3.8±0.20	7.8±0.31	3.5±0.07	3.5±0.04	3.4±0.11	4.2±0.05	40.4	80.8
	14 day	6.0±0.21	6.5±0.40	3.5±0.23	7.9±0.20	3.3±0.12	3.2±0.15	3.0±0.13	3.8±0.10	37.2	74.4
PSMK	Fresh	8.0±0.28	9.0±0.23	4.5±0.17	8.0±0.24	3.5±0.06	4.0±0.05	4.5±0.18	4.6±0.15	46.1	92.2
	7 day	8.0±0.15	8.0±0.19	4.2±0.33	8.2±0.23	3.2±0.13	4.2±0.07	4.1±0.08	4.5±0.10	44.4	88.8
	14 day	7.5±0.25	7.5±0.27	3.9±0.14	8.3±0.25	3.0±0.07	4.0±0.14	3.5±0.05	4.1±0.17	41.8	83.6
Tukey test	p-value	0.195	0.000*	.0.190	0.482	0.247	0.172	0.000*	0.214	-	-
	MSD	ns	2.023	ns	ns	ns	ns	0.991	ns		

The minimum significant difference (MSD) was considered at $\alpha \leq 0.05$, n = 20. *Significant differences, ns; nonsignificant differences.

Table S2. The general arrangement of collected data obtained during kefir beverage preparation used for the estimation of the correlation coefficient between the three kefir beverages irrespective of the storage period.

Time	Parameter		Cow milk kefir	Water sesame milk kefir	Permeate sesame milk kefir
Fresh	Component	TS	12.680	13.310	16.380
		Protein	3.200	2.940	3.140
		Lipids	3.100	5.240	5.890
		Sugars	3.370	1.510	4.890
		Ash	0.660	0.760	0.840
		TA	0.820	0.680	0.730
		pH	4.620	4.750	4.700
	Macro-element	Ca	112.050	220.150	255.000
		P	160.240	121.050	133.210
		K	100.120	53.110	66.640
		Na	37.370	40.270	44.180
		Mg	9.680	80.970	89.910
	Micro-element	Cu	0.010	0.870	3.510
		Fe	0.080	1.500	1.750
		Zn	0.330	2.050	2.340
		Mn	0.005	0.410	0.510
		Se	1.010	2.180	5.120
	Volatile compounds	Acetaldehyde	4.910	4.440	7.480
		Ethanol	0.071	0.129	0.105
	Physical properties	Viscosity (cP)	1.420	1.330	1.450
		$L*$	82.110	79.970	81.060
		$a*$	-4.101	-4.890	-4.091
		$b*$	16.210	14.410	16.270
		Chroma	16.720	15.220	16.780
		Hue angle (h°)	104.160	108.710	104.080
	Microbial group	Total bacteria	8.530	7.420	7.270
		Lactobacilli	8.170	6.350	7.180
		Lactococci	8.260	6.690	7.220
		Yeast	5.710	5.230	5.410
	Sensory evaluation	Homogeneous	9.000	7.500	8.000
		Color	9.500	7.500	9.000
		Consistency	4.700	4.200	4.500
		Slightly sour smell	8.100	7.500	8.000
		Slightly yeasty taste	4.000	3.700	3.500
		Refreshing taste	4.200	3.800	4.000
		Pleasant, fermented taste	4.800	4.000	4.500

Time	Parameter		Cow milk kefir	Water sesame milk kefir	Permeate sesame milk kefir
		Recommend buying	5.000	4.300	4.600
7 days	Component	TS	12.980	13.420	16.420
		Protein	3.260	2.970	3.210
		Lipids	3.110	5.270	5.910
		Sugars	3.250	1.350	4.720
		Ash	0.690	0.780	0.910
		TA	0.900	0.720	0.820
		pH	4.520	4.710	4.630
	Macro-element	Ca	110.320	241.110	241.020
		P	151.080	112.310	125.110
		K	89.210	50.170	65.110
		Na	37.120	38.180	44.010
		Mg	9.550	78.580	88.210
	Micro-element	Cu	0.010	0.820	3.240
		Fe	0.070	1.210	1.650
		Zn	0.310	2.170	2.220
		Mn	0.004	0.430	0.500
		Se	1.000	2.370	5.000
	Volatile compounds	Acetaldehyde	11.770	8.570	15.980
		Ethanol	0.104	0.145	0.135
	Physical properties	Viscosity (cP)	1.450	1.360	1.460
		$L*$	81.180	77.110	80.210
		$a*$	-4.621	-6.293	-3.168
		$b*$	17.250	14.450	17.430
		Chroma	17.860	15.760	17.720
		Hue angle (h°)	104.960	113.500	100.260
	Microbial group	Total bacteria	7.660	7.110	7.050
		Lactobacilli	7.650	5.770	6.750
		Lactococci	7.460	5.880	6.630
		Yeast	5.770	5.410	5.490
	Sensory evaluation	Homogeneous	8.500	7.000	8.000
		Color	9.000	7.200	8.000
		Consistency	4.300	3.800	4.200
		Slightly sour smell	8.300	7.800	8.200
		Slightly yeasty taste	3.500	3.500	3.200
		Refreshing taste	4.400	3.500	4.200
		Pleasant, fermented taste	4.700	3.400	4.100
		Recommend buying	5.000	4.200	4.500
14 days	Component	TS	13.180	13.530	16.560

Time	Parameter		Cow milk kefir	Water sesame milk kefir	Permeate sesame milk kefir
		Protein	3.270	3.050	3.220
		Lipids	3.100	5.260	5.900
		Sugars	3.050	1.210	4.510
		Ash	0.710	0.820	0.940
		TA	0.940	0.810	0.920
		pH	4.450	4.630	4.460
	Macro-element	Ca	114.210	253.020	258.230
		P	121.620	132.700	137.140
		K	90.110	52.920	70.240
		Na	35.120	44.190	45.000
		Mg	9.170	81.960	91.240
	Micro-element	Cu	0.010	0.850	3.470
		Fe	0.060	1.280	1.770
		Zn	0.300	2.340	2.450
		Mn	0.004	0.540	0.570
		Se	1.020	2.500	5.180
	Volatile compounds	Acetaldehyde	16.920	12.630	17.460
		Ethanol	0.139	0.247	0.215
	Physical properties	Viscosity (cP)	1.570	1.400	1.530
		L^*	81.150	76.050	81.180
		a^*	-4.688	-6.381	-3.171
		b^*	17.870	15.450	18.480
		Chroma	18.470	16.720	18.750
		Hue angle (h°)	104.660	112.410	99.700
	Microbial group	Total bacteria	7.860	7.310	7.180
		Lactobacilli	7.190	5.510	6.410
		Lactococci	6.860	5.640	6.210
		Yeast	5.810	5.460	5.570
	Sensory evaluation	Homogeneous	8.000	6.000	7.500
		Color	8.800	6.500	7.500
		Consistency	4.000	3.500	3.900
		Slightly sour smell	8.500	7.900	8.300
		Slightly yeasty taste	3.300	3.300	3.000
		Refreshing taste	3.900	3.200	4.000
		Pleasant, fermented taste	3.500	3.000	3.500
		Recommend buying	4.200	3.800	4.100

Table S3. The general arrangement of collected data of kefir grains used for estimation of the correlation coefficient between the three kefir grains irrespective of the storage period

Time	Parameter	CM-KG	WSM-KG	PSM-KG
Before incubation	Kefir grain biomass (g)	2.56	2.55	2.52
	pH	6.61	6.72	6.64
	Titratable acidity (%)	0.16	0.14	0.15
Fresh	Kefir grain biomass (g)	3.93	3.61	3.77
	pH	4.23	4.45	4.28
	Titratable acidity (%)	1.05	0.92	0.99
7 days	Kefir grain biomass (g)	4.17	4.03	4.05
	pH	4.12	4.23	4.18
	Titratable acidity (%)	1.13	1.04	1.05
14 days	Kefir grain biomass (g)	4.23	4.11	4.16
	pH	4.03	4.15	4.02
	Titratable acidity (%)	1.14	1.11	1.15

Cow milk kefir grains (CM-KG), water sesame milk kefir grains (WSM-KG), and permeate sesame milk kefir grains (PSM-KG).

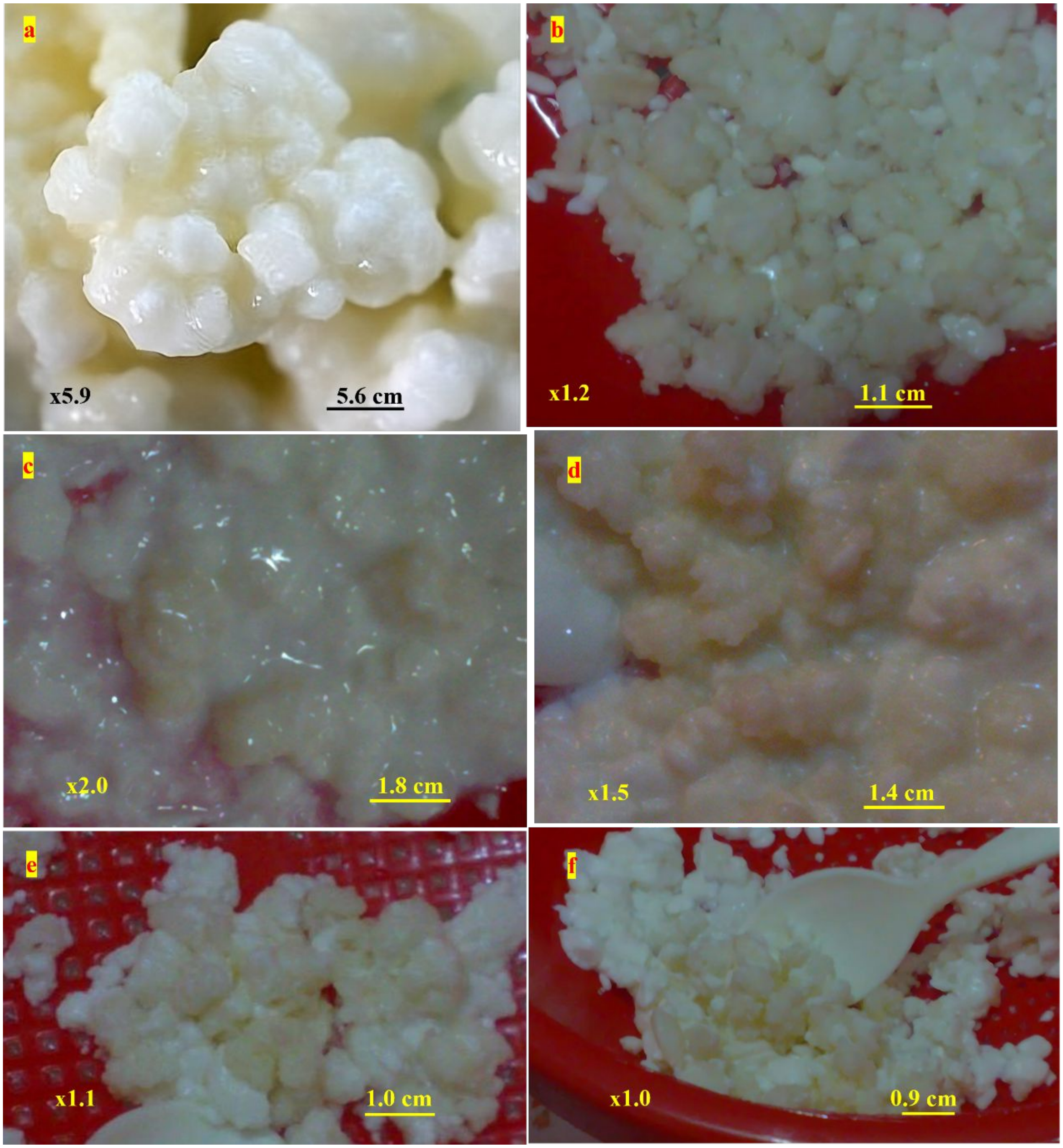

Figure S1. Zoom-in view of kefir grain morphology developed in cow milk (a, and b), water sesame milk (c, and d), and permeate sesame milk (e, and f). These photos were taken by one of the authors during the manufacturing in the laboratory.

(a)

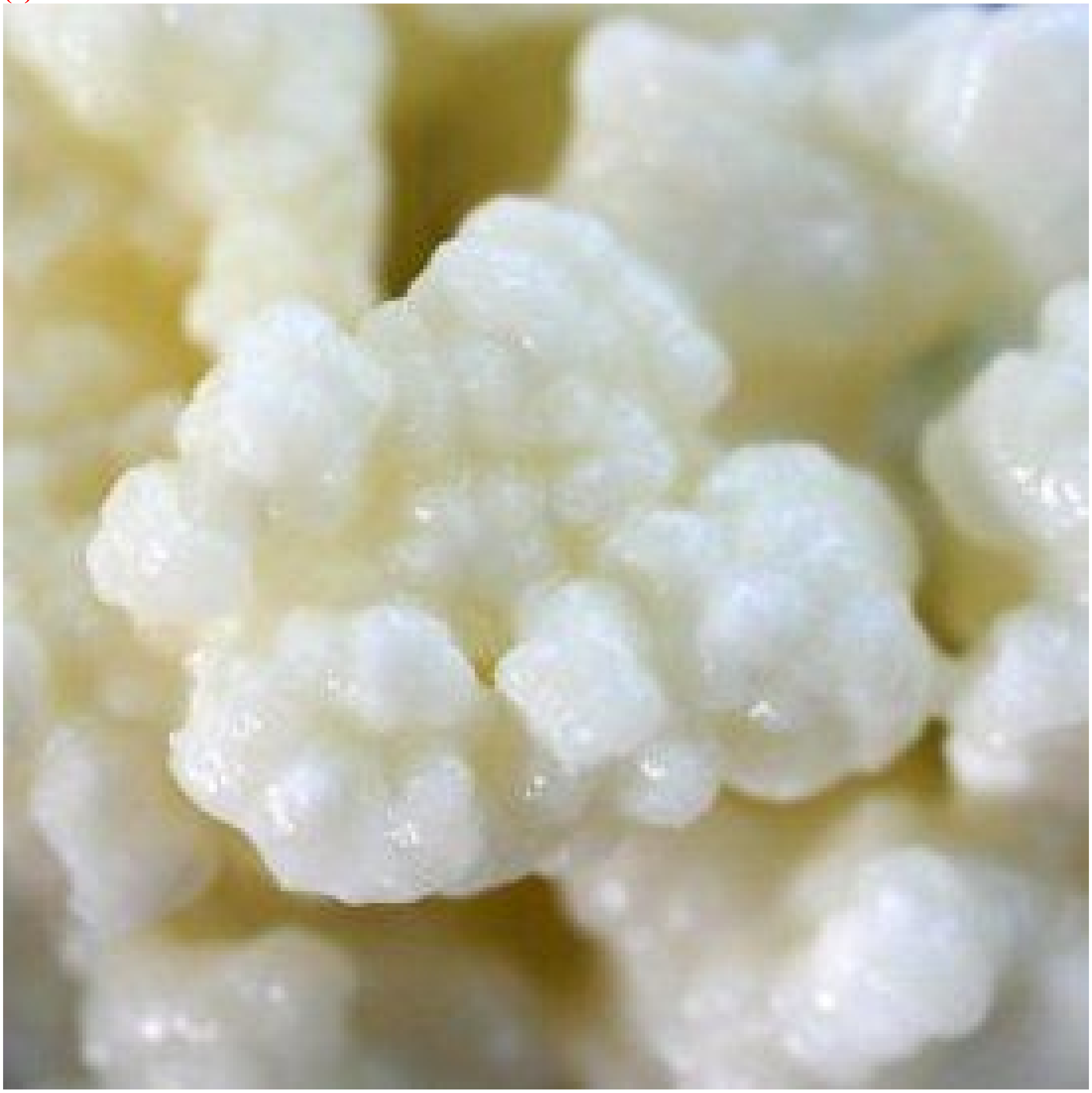

(b)

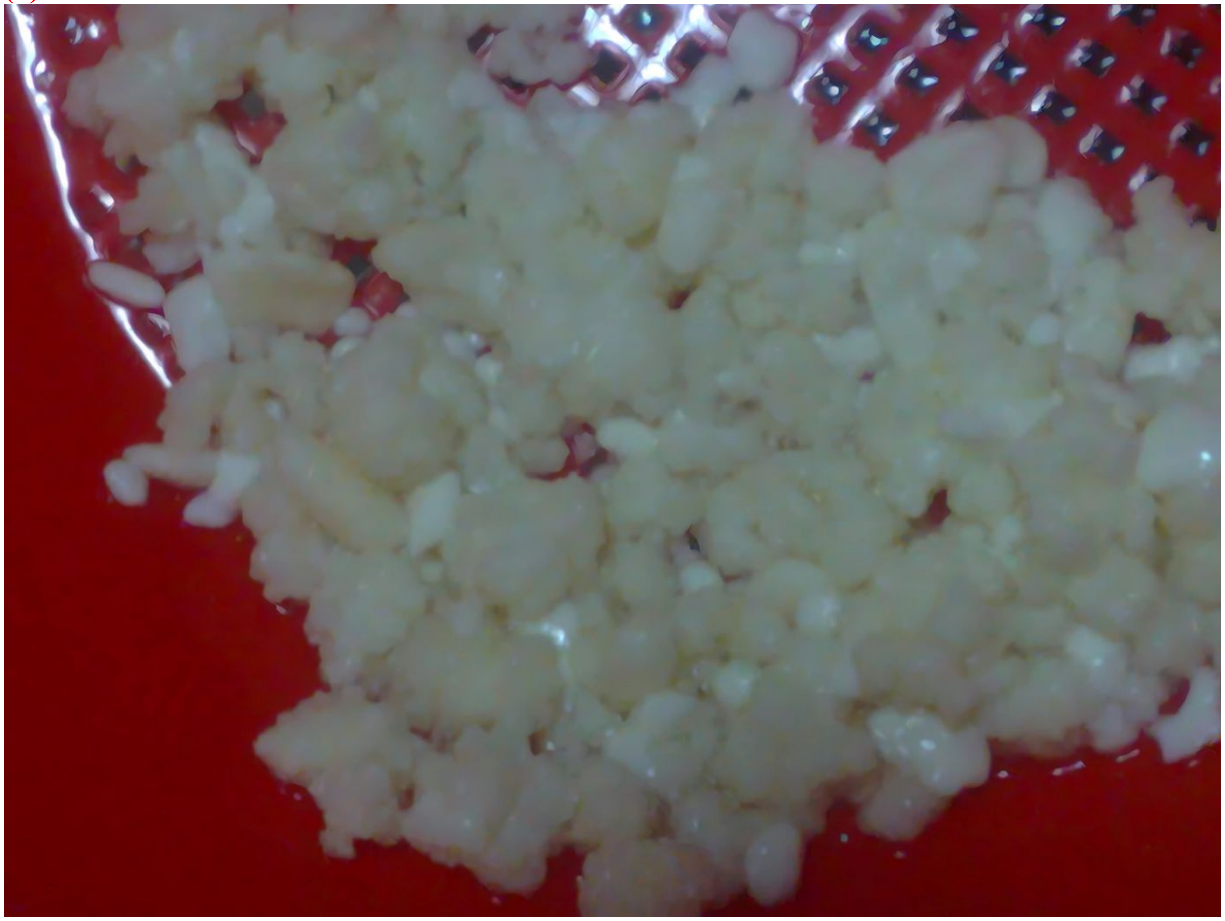

Figure S2. Zoom-in view of kefir grain morphology developed in cow milk. These photos were taken by one of the authors during the manufacturing in the laboratory.

(c)

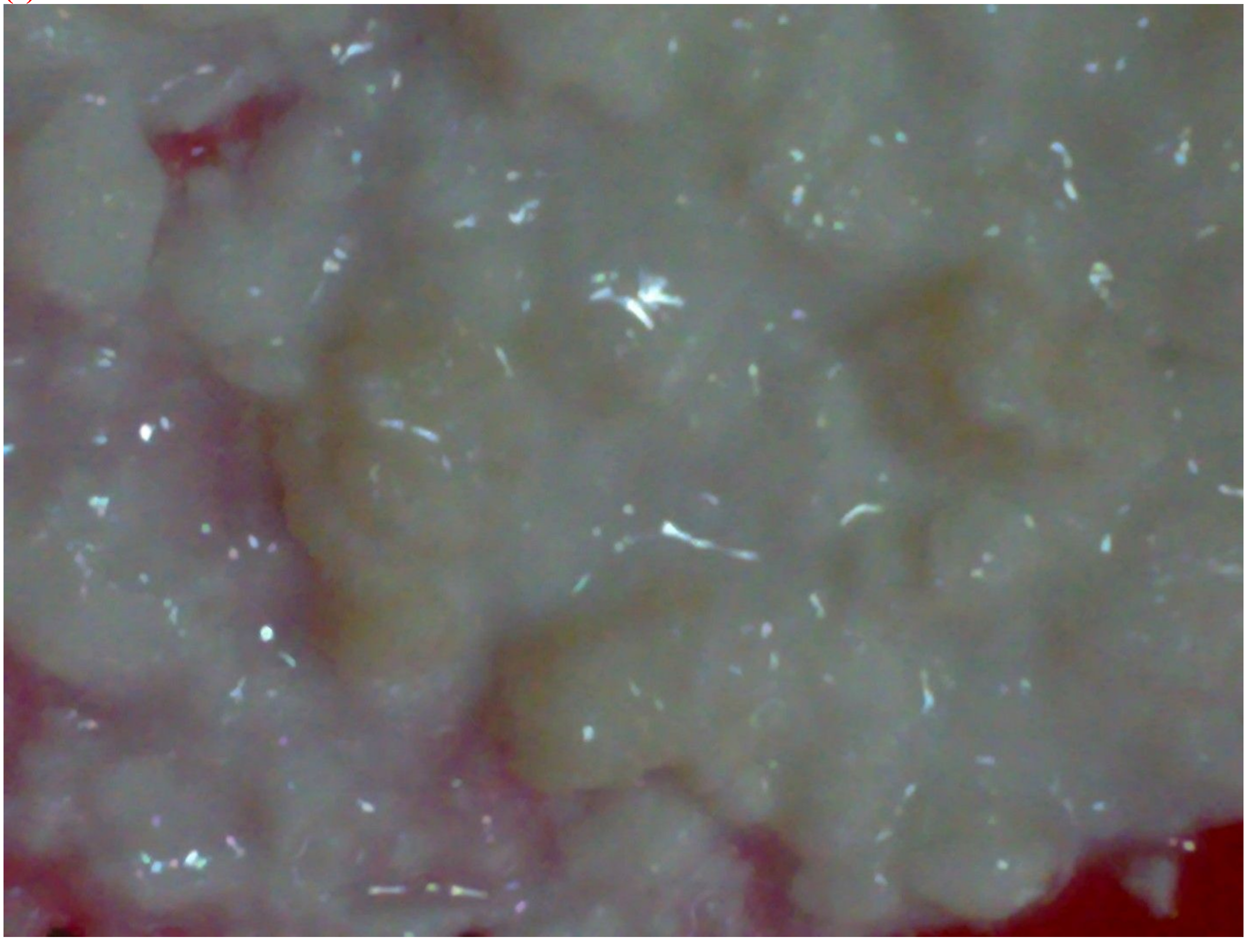

(d)

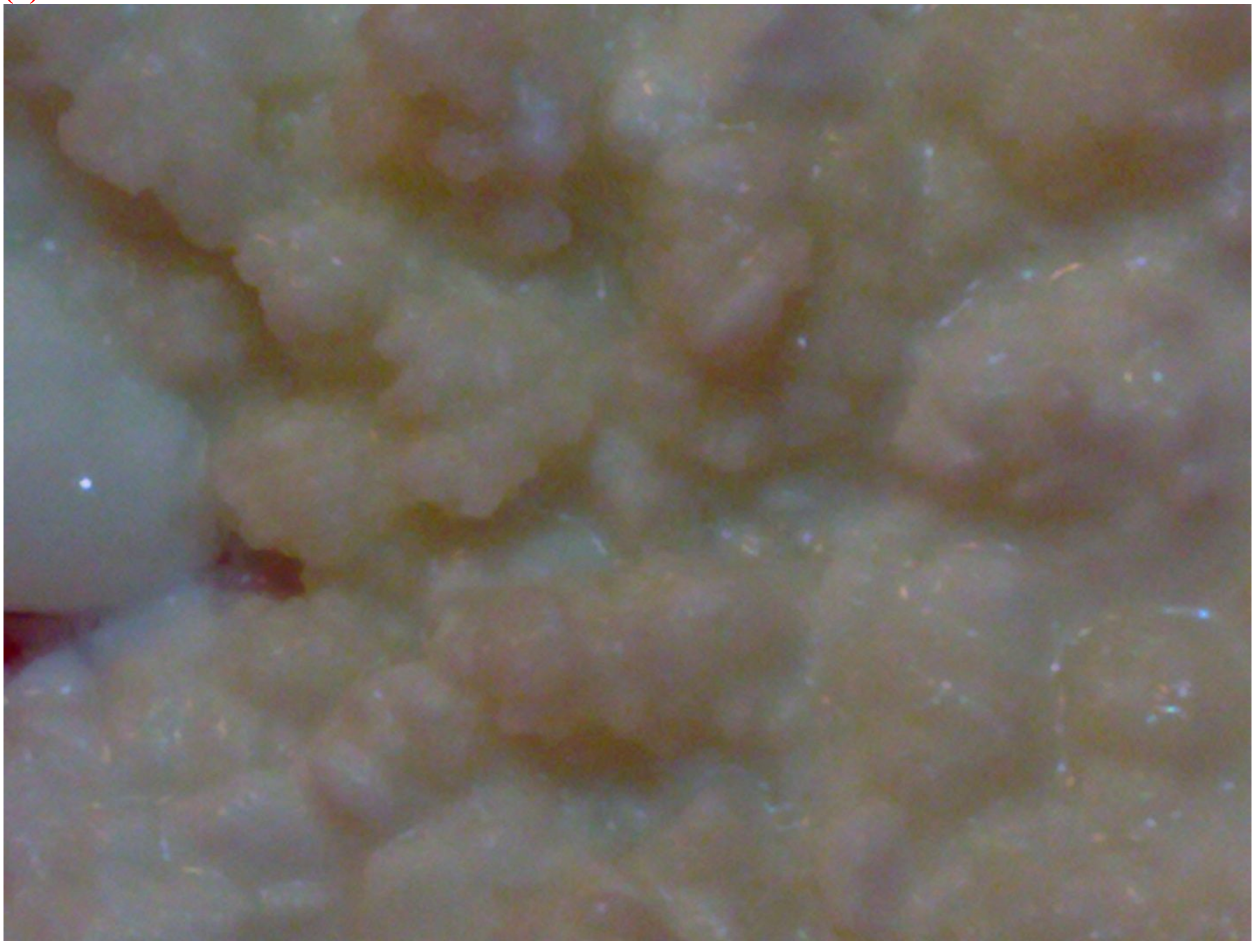

Figure S3. Zoom-in view of kefir grain morphology developed in water sesame milk. These photos were taken by one of the authors during the manufacturing in the laboratory.

(e)

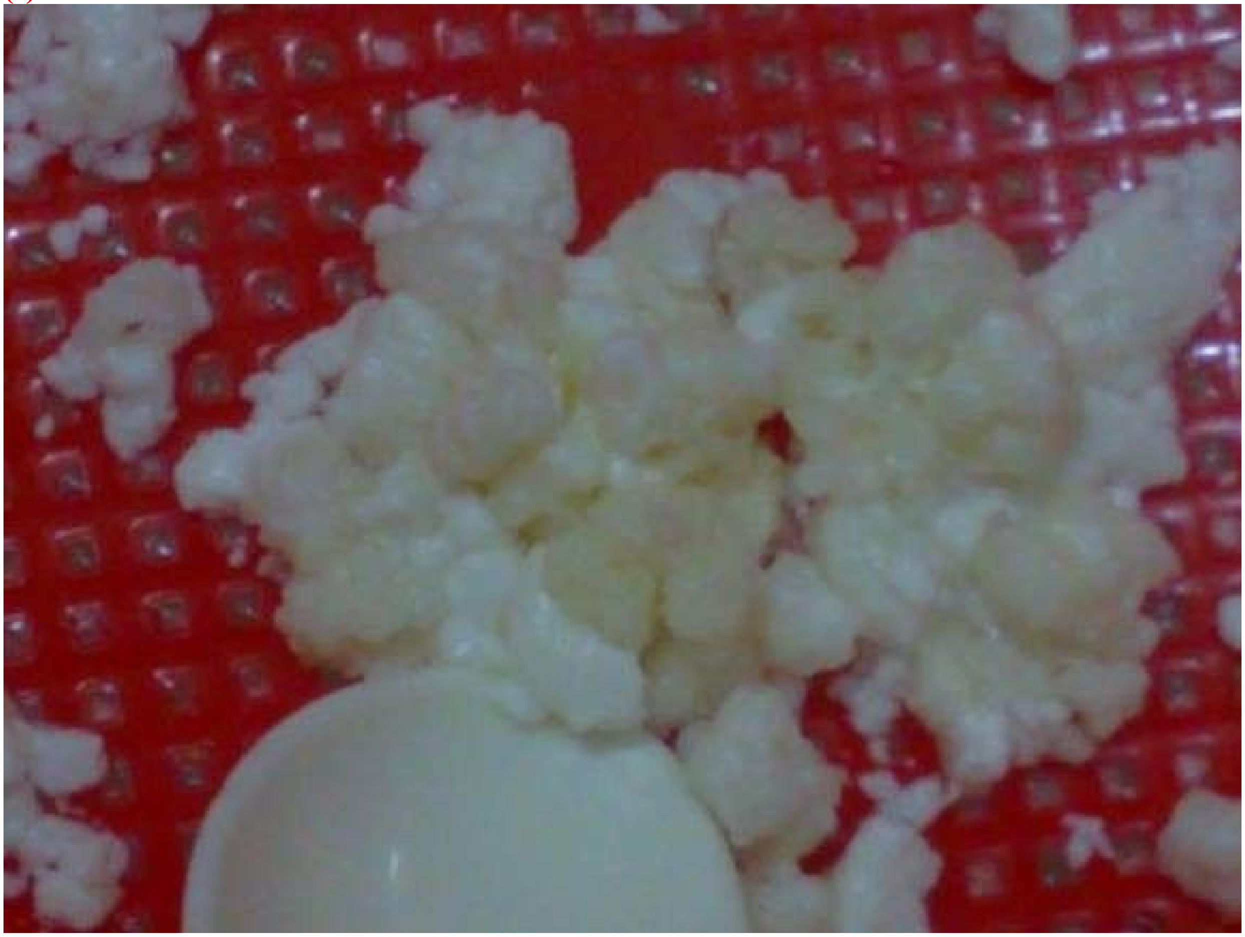

(f)

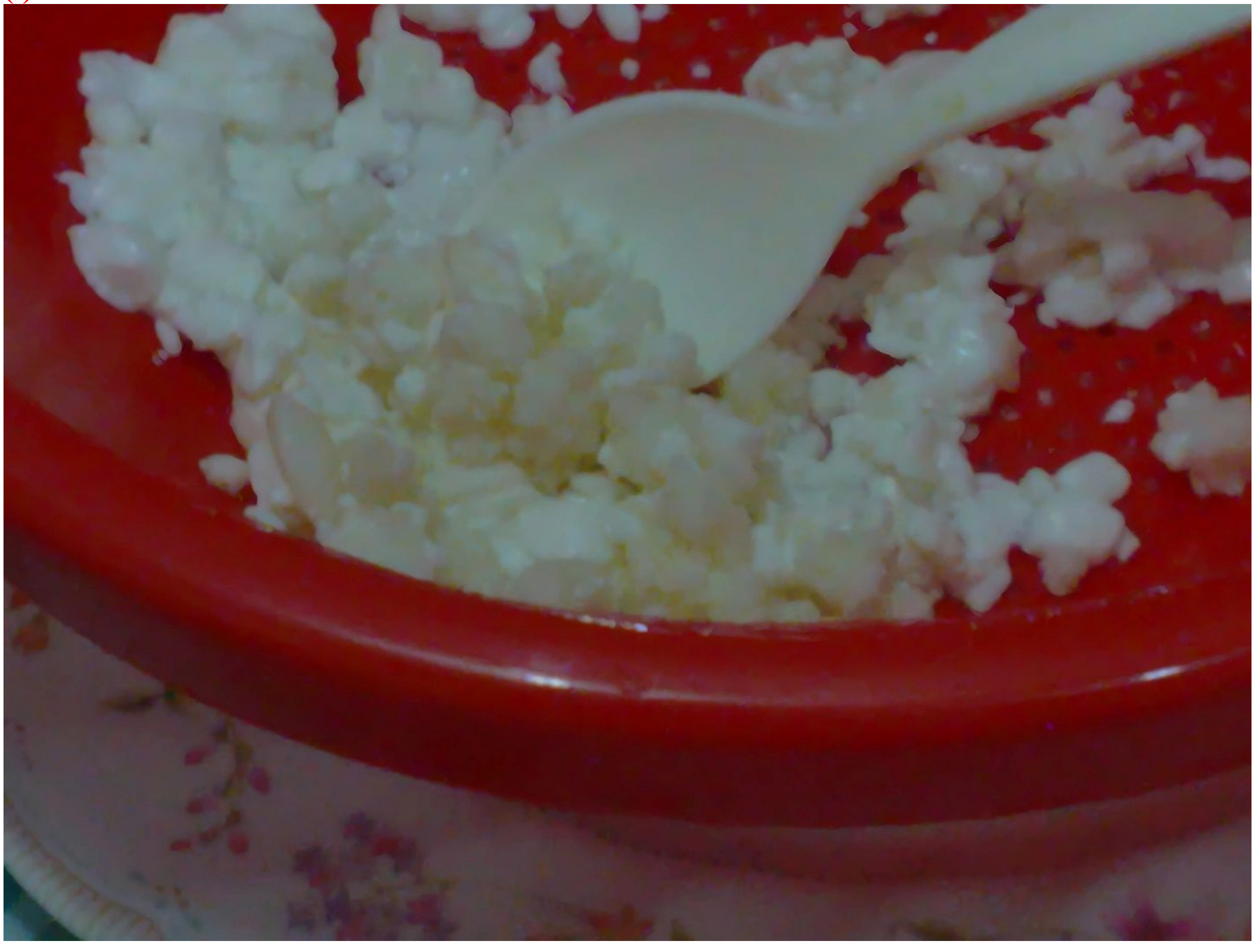

Figure S4. Zoom-in view of kefir grain morphology developed permeate sesame milk. These photos were taken by one of the authors during the manufacturing in the laboratory.

(a)

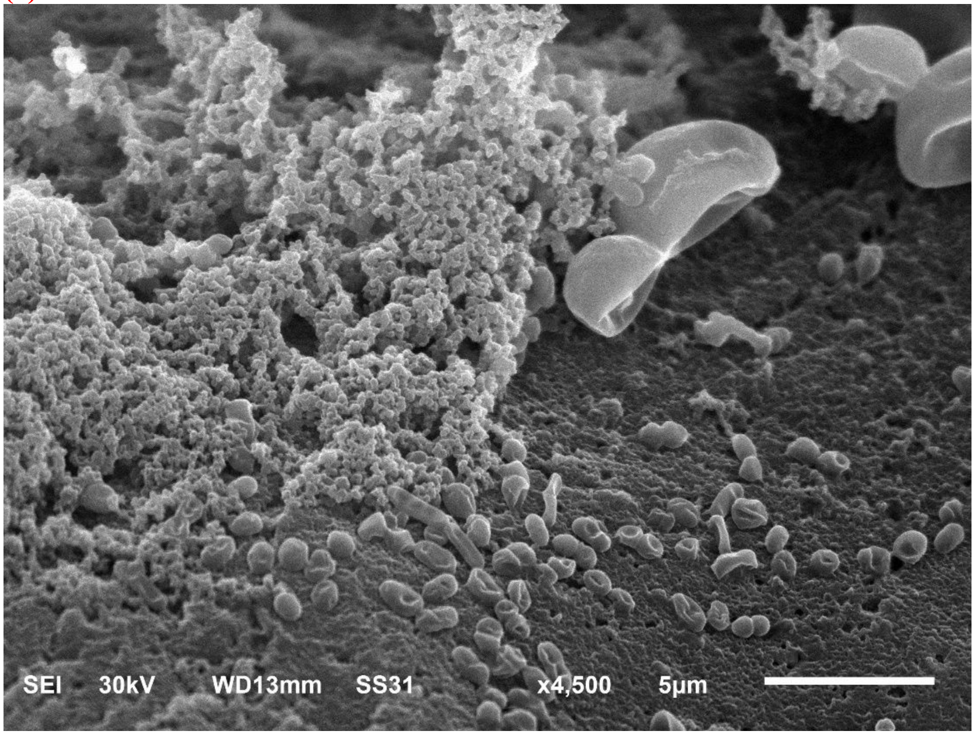

(b)

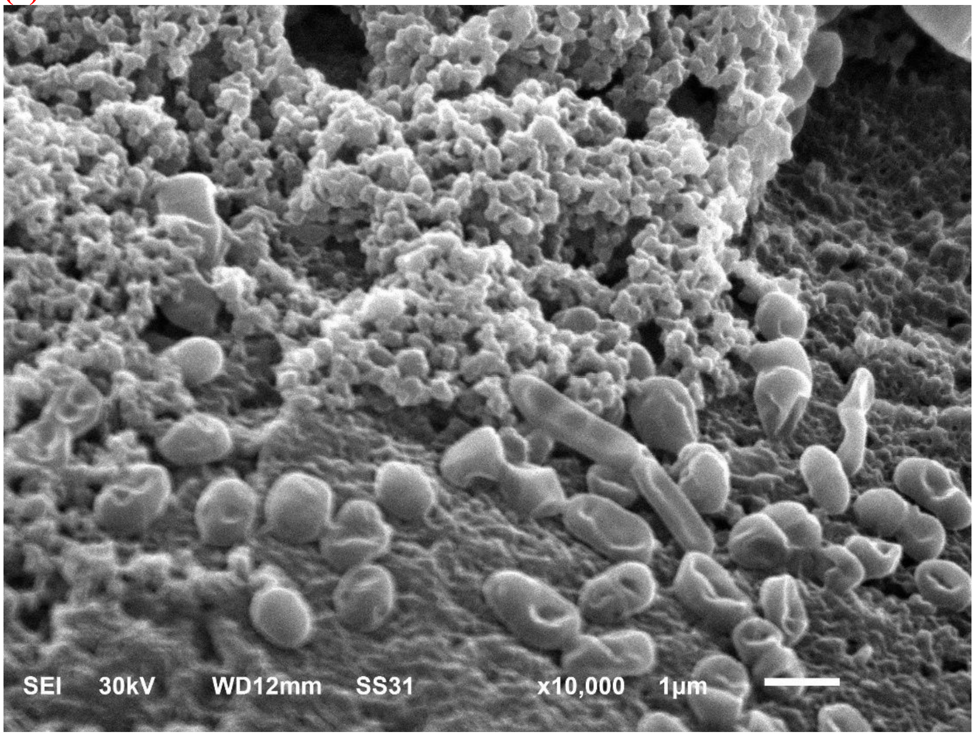

Figure S5. SEM image of kefir grain, developed in cow milk medium, showcasing the interconnected network of microbial cells within the kefiran matrix at magnification of 4500x (a), and 10000 (b).

(a)

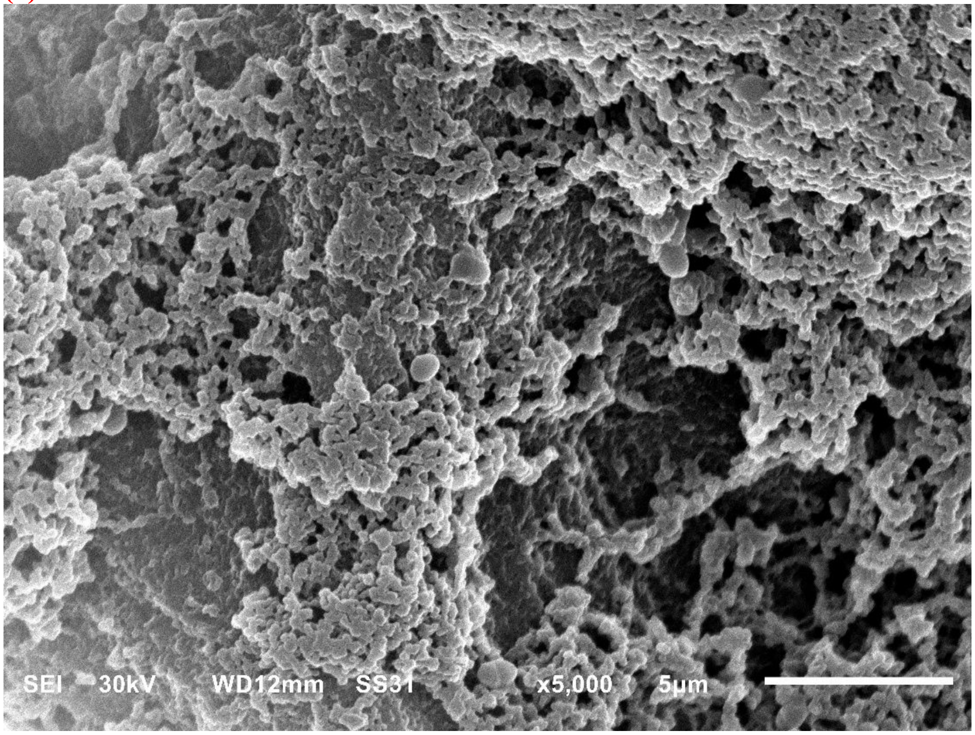

(b)

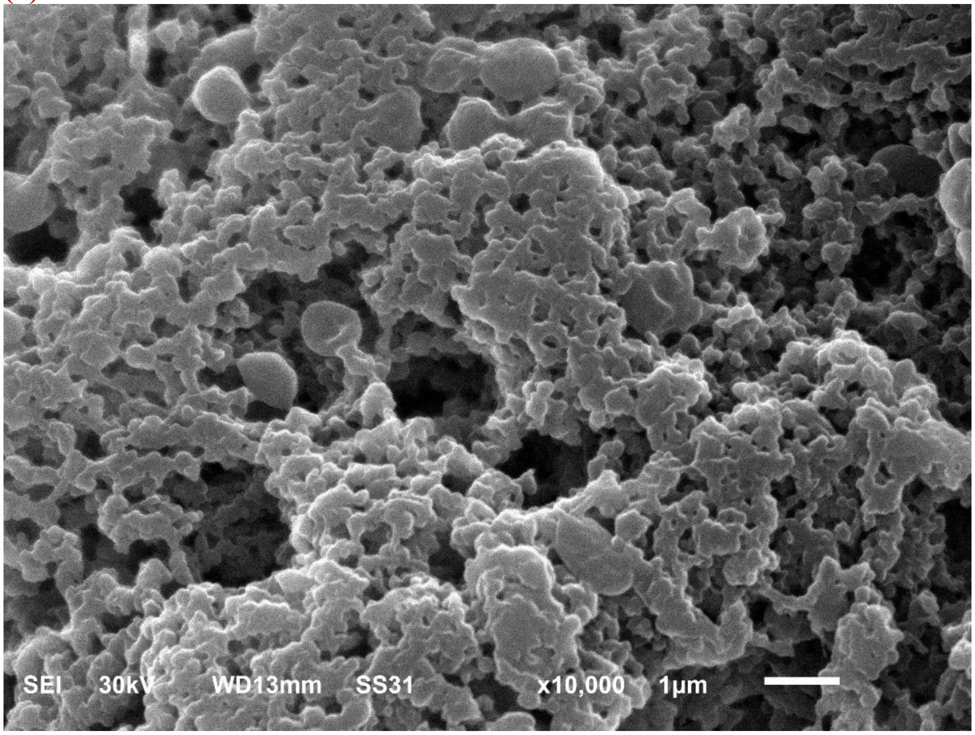

Figure S6. SEM image of kefir grain, developed in WSM medium, showcasing the interconnected network of microbial cells within the kefiran matrix at a magnification of 5000x (a), and 10000 (b).

(a)

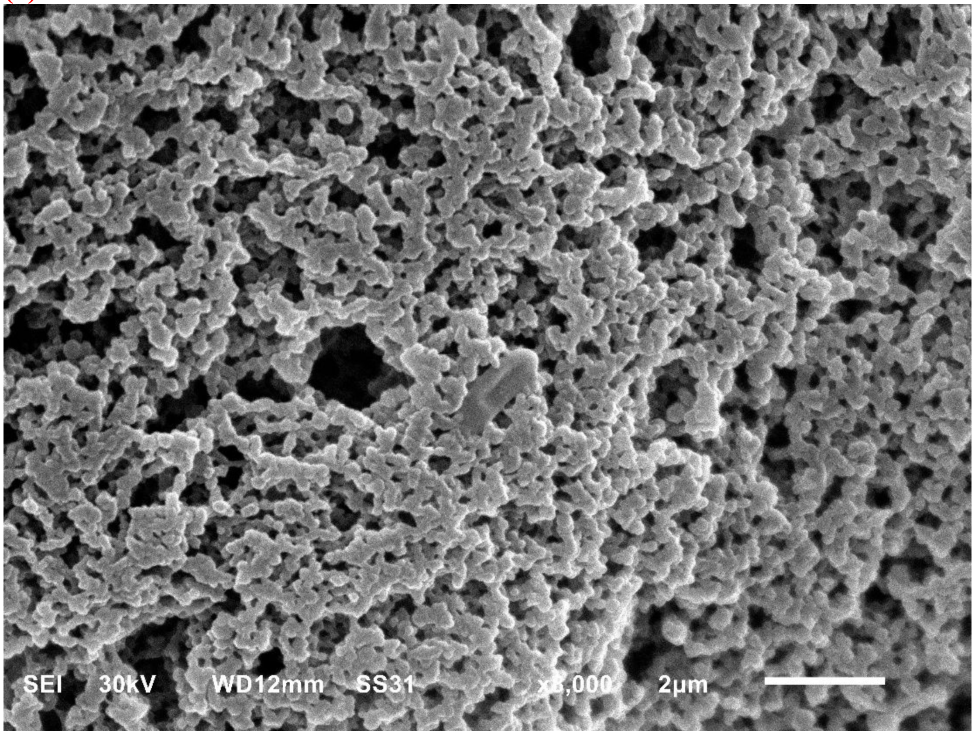

(b)

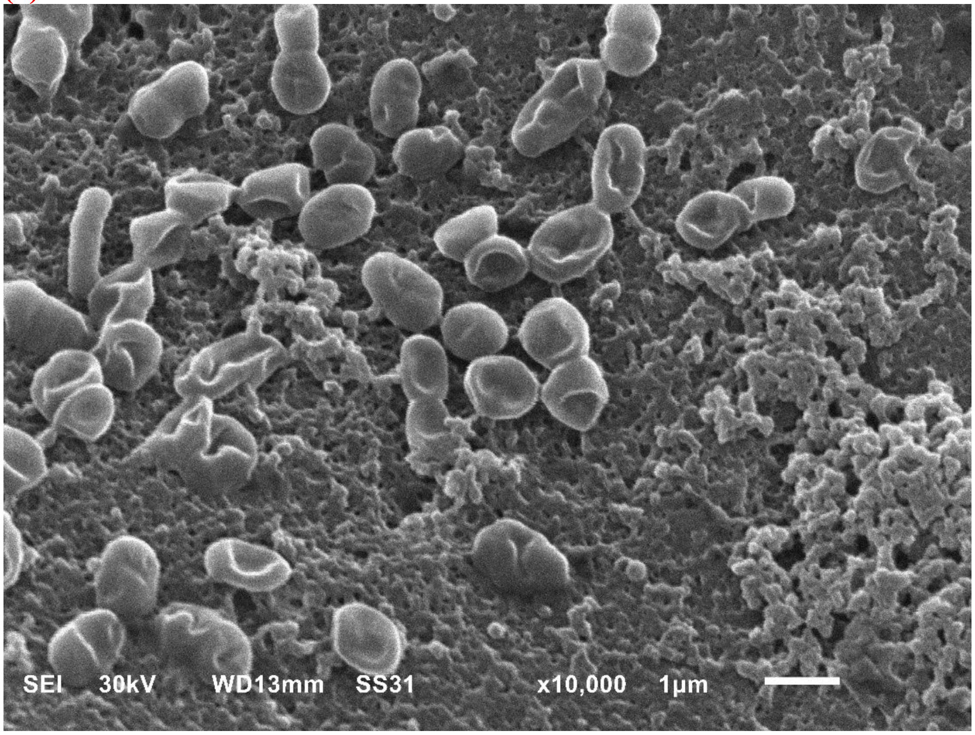

Figure S7. SEM image of kefir grain, developed in PSM medium, showcasing the interconnected network of microbial cells within the kefiran matrix at a magnification of 8000x (a), and 10000 (b).
